# Ecology of a widespread large omnivore, *Homo sapiens*, and its impacts on ecosystem processes

**DOI:** 10.1002/ece3.5049

**Published:** 2019-09-11

**Authors:** Meredith Root‐Bernstein, Richard Ladle

**Affiliations:** ^1^ Section for Ecoinformatics & Biodiversity, Department of Bioscience Aarhus University Aarhus Denmark; ^2^ Institute of Ecology and Biodiversity Santiago Chile; ^3^ UMR Sciences pour l'Action et le Développement, Activités, Produits, Territoires INRA, AgroParisTech, Université Paris‐Saclay Thiverval‐Grignon France; ^4^ Center of Applied Ecology and Sustainability (CAPES) Santiago Chile; ^5^ School of Science and Health Federal University of Alagoas Alagoas Brazil; ^6^ School of Geography and the Environment Oxford University Oxford UK

**Keywords:** defaunation, hominin, hunter‐gatherer, interspecific comparison, intraspecific variation, omnivory, taxon substitution

## Abstract

1. Discussions of defaunation and taxon substitution have concentrated on megafaunal herbivores and carnivores, but mainly overlooked the particular ecological importance of megafaunal omnivores. In particular, the *Homo *spp. have been almost completely ignored in this context, despite the extinction of all but one hominin species present since the Plio‐Pleistocene. Large omnivores have a particular set of ecological functions reflecting their foraging flexibility and the varied disturbances they create, functions that may maintain ecosystem stability and resilience. Here, we put the ecology of *Homo sapiens* in the context of comparative interspecific ecological roles and impacts, focusing on the large omnivore guild, as well as comparative intraspecific variation, focusing on hunter‐gatherers.
2. We provide an overview of the functional traits of *H. sapiens*, which can be used to spontaneously provide the functions for currently ecologically extinct or endangered ecosystem processes. We consider the negative impacts of variations in *H. sapiens* phenotypic strategies, its possible status as an invasive species, and the potential to take advantage of its learning capacities to decouple negative and positive impacts.
3. We provide examples of how practices related to foraging, transhumance, and hunting could contribute to rewilding‐inspired programs either drawing on hunter‐gatherer baselines of *H. sapiens*, or as proxies for extinct or threatened large omnivores. We propose that a greater focus on intraspecific ecological variation and interspecific comparative ecology of *H. sapiens* can provide new avenues for conservation and ecological research.

## Graphical abstract

The ecology of humans is usually considered separately and often using different concepts to those used for other species. We propose an ecological approach to humans based on the joint consideration of intraspecific variation and interspecific comparison. We look at inter‐ and intraspecific variation and similarity in ecological roles of hunter‐gatherer humans, other hominins, and other large omnivores.

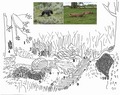

## 1 INTRODUCTION

In this paper, we take the unusual position of talking about *Homo sapiens* in ecological terms in exactly the same way that an ecologist would talk about nonhuman species. We do so because we have observed that *H. sapiens* is systematically considered within the ecological literature as an exception to normal ecological processes. Specifically, ecologists use different terminology to write about *H. sapiens*, generating a unique set of hypotheses and predictions. Indeed, it is almost as though *H. sapiens* were not an animal (for an explicit statement of this see Ellis, 2015). One could engage in a philosophical debate about whether the unique things *H. sapiens* does eclipse and negate the ways in which it is an animal, ecologically speaking. However, we find this approach overly conceptual and, instead, adopt an empirical, data‐based approach. There are already many ways of researching *H. sapiens*. We would like to contribute an additional, complementary, form of academic discourse about *H. sapiens,* centered modestly on the ecological processes in which *H. sapiens* may participate. Although we are not the first to make many of these points (see Bliege Bird & Nimmo, 2018; Castilla, 1999), this perspective remains challenging to address and develop.

**Figure 1:**
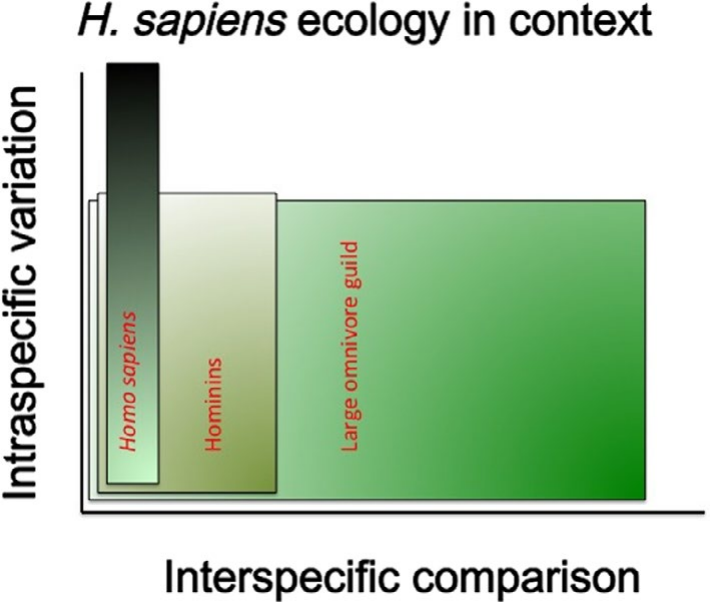
The framework of interspecific comparison and intraspecific variation in which we position this paper. The axes are not scaled. The overlap shown between *Homo sapiens* and other species' ecologies is not intended to be to scale or to make a quantitative claim about how much overlap there is

Perhaps the most central and most challenging issue is addressing both interspecific and intraspecific considerations. We attempt this through a comparative framework (Figure 1). At the interspecific level, we present the argument that there is a large range of overlap between *H. sapiens* ecology and the ecology of nonhuman species, which allows us to discuss *H. sapiens* in ecological terms. Our challenge in this paper is to examine the ways in which *H. sapiens* can be a subject of ecological enquiry, while not antagonizing researchers who either believe (a) *H. sapiens* is incomparable to nonhuman species because it has proved itself to be evolutionarily unique and any discussion must give priority to all of these differences (primarily ecologists); or (b) *H. sapiens* is infinitely more variable and flexible than ecology is willing to account for, and any discussion must therefore consider all of these variations (primarily anthropologists). Indeed, within ecology there may be a tendency to give relatively little emphasis to intraspecific variation in reproductive life‐histories, foraging strategies and diets, physiology, development, and social organization, such that the discovery of the importance of such variation is noteworthy and surprising (e.g., in animal ecology see Ford et al., 1997; Hebblewhite & Merrill, 2007, 2009; Montgomery et al., 2018; Putman & Flueck, 2011). We also note that there is no equivalent general term within ecology and evolution comparable to the terms “lifeway,” “livelihood,” or “subsistence strategy,” except possibly “phenotype,” in the sense of the expression of genes and epigenetic factors, and their plastic interactions with the environment, including development and physiology, behaviors, and morphology. We will use the term phenotype in this broad sense (not limited to gene expression as morphology) in the rest of the paper (Jablonka & Lamb, 2007; Piersma & Van Gils, 2011; West‐Eberhard, 1989).

For the purposes of this paper, we deliberately generalize about *H. sapiens* in exactly the same way that ecologists generalize about nonhuman species. This is intended as a provocation: What happens, intellectually, to our understanding of *H. sapiens* ecology if we generalize in an ecological style, rather than particularizing in an anthropological style (see Gremillion, Barton, & Piperno, 2014)? Generalizing in an ecological style specifically does not mean that we are following in the footsteps of other ecologists, who claim about *H. sapiens*, for example, that their impacts on the environment are “not biological” (Ellis, 2015) and that the paleohistory of their impacts on the global environment constitutes a “single event” (Chase‐Dunn & Lerro, 2013) without significant intraspecific variation in those impacts (Ellis, 2015). Indeed, our claim is that, far from being “romantic” or naive to view *H. sapiens* as an animal (Ellis, 2015), it is rather eminently scientific and rational: It is a removal of the anthropocentric exceptionalism bias and thus a claim to treat all data the same without prejudice. We hope to raise questions as to what forms of knowledge are made available to or occluded from ecological enquiry by treating species (or some species, but not others) at the intraspecific level as either uniform and phenotypically inflexible in their interactions with the environment, or vice versa, as so variable and flexible as to defy ecological generalizations about their interactions with the environment. We propose that valid ecological generalizations about all species would take into account both inter‐ and intraspecific variation.

We believe that taking a provocatively recentered view of *H. sapiens* ecology is important for at least two reasons: (a) A move away from an implicit position of human exceptionalism can help form new interdisciplinary connections based on commonalities between *H. sapiens* and nonhuman species across the life and environmental sciences; (b) the world can be different because it already is (Gibson‐Graham, 2006): as suggested by this famous claim, recognizing hidden realities can open up new bases for future action—here, we are thinking specifically of sustainability and conservation approaches. To again cite Ellis (2015), only because it is exceptionally explicit, the orthodox ecological view is that *H. sapiens* is enacting its “single event” destiny, following a unidirectional path of cultural development without significant intraspecific variation in its ecological impacts, leading to global destruction. Though many ecologists repeatedly claim that if only we understood the relevant variables better we could engineer positive socio‐ecological outcomes, one honestly has to ask whether their own reasoning provides any theoretical basis for their claim that the future can be radically different from all of the past (which they claim forms a single trajectory). Shifting the frame away from this exceptionalist discourse that denies significance to both interspecific comparison and intraspecific variation, toward an empirically based attention to the range of possible ecological roles and impacts of *H. sapiens,* opens up new theoretical grounds for practical conservation work.

We opened the paper by stating that we want to move from overly conceptual debates to empirical data, so why are we now calling for a critical shift in conceptualization? Our claim is that this shift will refocus how we attend to empirical data. A good example of how a shift in perspective can reveal unexpected empirical observations and new research directions is Bond and Keeley (2005). This review paper reframed fire as a type of herbivory, leading to novel insights about the role of fire in ecosystems and in interactions with (animal) herbivores, its “naturalness,” and its role in evolution, with important impacts on the field of fire ecology. Another example comes from Marx's environmental writings (Foster, 1999). Marx had the insight, during the English soil fertility crisis of the mid‐1800s, that the migration of peasants to urban industrial centers meant that nutrients were exported, as food, from agricultural lands to cities, and then not returned to the ecosystems of origin since urban human feces were channeled through sewage systems to the sea, leading to a gradual loss in soil fertility. This early reframing of agriculture, industrialization, and social change as nutrient dynamics contributed to Marx's critique of capitalism. More prosaically, Root‐Bernstein and Svenning (2018) suggest that new approaches to managing overuse of nature tourism trails could emerge from refocusing this issue on the ecology of how and when *H. sapiens* and small mammal trails, which they demonstrate have similar effects, positively affect plant biodiversity.

Here, we consider a reframing of *H. sapiens* as an ecological actor through the lens of its potential roles in ecological restoration. We first discuss the ecological theoretical context in which we frame this discussion; we then consider the phenotypes and niches of *H. sapiens*, other Homo species, and large omnivores, and their possible ecological impacts. Finally, we consider some possible impacts of *H. sapiens* as a taxon substitute for the considered ecological impacts where they have been lost, and provide a general overview of approaches to doing so. This subject is so vast that we do not claim to have written a review paper; it is rather the outline for a giant interdisciplinary review project that could take years, if not decades.

## 2 CONTEXT OF ECOLOGICAL THEORY

Species extinctions are one of the most urgent conservation threats today. A large “extinction debt” is thought to already exist, with many species possibly fated to disappear (Kuussaari et al., 2009; Tilman, May, Lehman, & Nowak, 1994). Long before species entirely disappear, however, their ecological functions may go extinct (Janzen, 2001; Valiente‐Banuet et al., 2015). The loss of ecological functions can result when a species is at too low a density or too few in number to significantly carry out an ecological process. In biodiverse ecosystems, multiple species with similar ecological roles are expected to be functionally redundant, acting as buffers so that environmental perturbations and ecological and/or evolutionary extinctions do not immediately impact the ecosystem (Tylianakis, Laliberté, Nielsen, & Bascompte, 2010). Nevertheless, many ecological communities eventually suffer “ecological downsizing” in which successively smaller species are extirpated or go extinct (Norkko, Villnas, Norkko, Valanko, & Pilditch, 2013; Pérez‐Méndez, Jordano, & Valido, 2015). This defaunation contributes to degradation by altering the spatiotemporal patterns of many ecological processes (Bruun & Fritzbøger, 2002; Dirzo et al., 2014; Root‐Bernstein et al., 2016). Contemporary defaunation is not the only baseline for restoration, however. Various baselines for defining a functioning ecosystem may be proposed, since the dramatic loss of species with key ecological roles has been ongoing since the late Pleistocene in many areas (Donlan et al., 2006; Martin, 1970). Loss of ecological functions due to Pleistocene extinctions may continue to affect current ecosystem dynamics (Gill, Williams, Jackson, Lininger, & Robinson, 2009; Guimarães, Galetti, & Jordano, 2008; Johnson, 2009; Martin, 1970; Rule et al., 2008).

At least two approaches exist to reverse ecological extinction. One is to reintroduce missing species to a sufficient population size that their ecological functions are restored (Seddon, Griffiths, Soorae, & Armstrong, 2014). However, when the extinct ecological functions were only carried out in a particular ecosystem by a species that is now evolutionary extinct, functionally equivalent species may be introduced to restore the ecological function, known as “taxon substitution” (Searcy, Rollins, & Shaffer, 2016). Rewilding has developed as one conservation and restoration approach that may use either reintroduction or taxon substitution to restore functions that underlie ecological processes (Lorimer et al., 2015; Root‐Bernstein, Galetti, & Ladle, 2017; Svenning et al., 2015). Implicit in the rewilding approach is the goal or intention to establish self‐managing ecosystems whose biodiversity and ecosystem processes are maintained spontaneously and endogenously by the species and abiotic elements in the ecosystem (Lorimer et al., 2015; Root‐Bernstein et al., 2017; Svenning et al., 2015). Increasing recognition of ecological extinction, and attempts to reverse it through reintroductions and taxon substitutions, has contributed to a recent focus on functional ecology, and on the ecology of many extinct and endangered large and medium‐sized mammals (Andriuzzi & Wall, 2018; Galetti, Pires, Brancalion, & Fernandez, 2017; Johnson et al., 2018; Naundrup & Svenning, 2015; Root‐Bernstein & Svenning, 2016; Sanderson et al., 2008).

However, we observe a rather startling set of underdeveloped areas in this literature: What are or were the ecological roles of *H. sapiens*, its recently extinct congeneric clade members, and extant guild members (omnivores)? In what follows, we consider the phenotypes and ecological extinctions of these species, their functional ecology, and possible ecological impacts, and consider some approaches to restoring potentially missing ecological functions attributable to them.

## 3 THE PHENOTYPES AND ECOLOGICAL EXTINCTIONS OF *HOMO SAPIENS*, HOMININS, AND LARGE OMNIVORES

To develop our comparative framework, we first describe the phenotypes in the broad sense of *H. sapiens*, then those of the extinct species from the genus Homo, and finally those of other large omnivores that can be considered to form an ecological guild including *H. sapiens*.

The genus *Homo* includes one extant species (*Homo sapiens *Linnaeus) listed globally as Least Concern (IUCN Red List, accessed 2014). *Homo sapiens* has the largest distribution of any terrestrial mammal, has populations in many protected areas, and is regulated under Appendix II of CITES as well as a number of species‐specific international treaties and national laws. There is debate about its native status across its range, implications for which we discuss below. *H. sapiens* is megafaunal by some definitions, at >44 kg (Martin, 1984; Stuart, 1991). It is an omnivore, defined as acting as both a predator and an herbivore (Bonhommeau et al., 2013; Kratina, LeCraw, Ingram, & Anholt, 2012). *Homo sapiens* is also a habitat generalist that uses cultural innovations unusually extensively in the acquisition and processing of resources, and is notable for the exceptional magnitude and breadth of its niche constructing effects (Boivin et al., 2016; Burnside et al., 2012; Ellis, 2015; Rowley‐Conwy & Layton, 2011; Sullivan, Bird, & Perry, 2017). Indeed, due to its extensive and pervasive role in many ecosystems *Homo sapiens* has been argued to be a “hyperkeystone” species (Worm & Paine, 2016).

*Homo sapiens* populations have at least two distinct phenotype clusters, not in the morphological sense, but in the broader sense of life‐history strategies, foraging, diet and physiological strategies, and social organizations: the hunter‐gatherer strategy and the agriculture strategy (Gremillion et al., 2014; Kelly, 1995). Here, we include pastoralism within agriculture (Diamond, 2002; Grigg, 1974), although some researchers consider this as a separate strategy. These two (or three) strategies form the extreme ends of a gradient, with agriculturalists often performing some hunting or gathering activities, and hunter‐gatherers often performing some plant and animal tending (Harris, 2012). As we move toward the agriculture strategy, ecological interactions become, in general, limited to a smaller number of interacting species, higher intensity, more predictable (less flexible) in space and time, and involving a greater number of less flexible partitions in ecological roles between individuals, compared to a hunter‐gatherer strategy within the same habitat (Gepts et al., 2012). Both strategies involve populations at low density engaged in a highly variable suite of disturbance activities, mutualisms, and ecosystem engineering/niche construction activities. The hunter‐gatherer strategy, while varying widely, can be characterized by a common specialization of males as functional carnivores, while females remain functional omnivores, that is, they both hunt game and gather animal, plant, and fungal foods (Allendorf & Hard, 2009; Bleige Bird, Codding, & Bird, 2009; Estioko‐Griffin, 1986; Kelly, 1995). Another way to describe this is that males tend to specialize in high‐variance forms of foraging that result in unpredictable “windfall” harvests; this usually means hunting large mobile prey (Bleige Bird et al., 2009). The agriculturalist strategy, which emerged only in the last 10,000 years (Gremillion et al., 2014), generally also includes sex‐based differentiation in ecological roles (though what the roles are varies), as well as individuals who, though they depend on agriculture sensu stricto for food, have facultative behavioral specializations that may not involve food production, and inhabit complex, ecosystem‐engineered and constructed habitats at high population densities. Hunter‐gatherer and agriculturalist *H. sapiens* often co‐occur (Bharucha & Pretty, 2010).

*Homo sapiens* is well known to demonstrate significant phenotypic plasticity and environment–phenotype interactions (Collard & Wood, 2007; Gilbert & Epel, 2009; Ross, Moate, Marett, Cocks, & Hayes, 2013; Rowley‐Conwy & Layton, 2011). All species have some combination, which varies across species, of fixed species‐specific genetic control over the phenotype, and epigenetic and environmentally induced variation and plasticity (Gilbert, 2001; Tinbergen, 1989). For *H. sapiens,* there are debates over how this integration works (Hawkes & Coxworth, 2013; Jones, 2015; Kelly, 1995; Loo, Hawkes, & Kim, 2017; Marlowe, 2005; Svizzero & Tisdell, 2015). Though sometimes unproductive, due to the difficulty of distinguishing between the nature of the different influences on the phenotype in the broad sense, these debates put a particular emphasis on within‐species variation, which, as we noted in the Introduction, is sometimes underappreciated in ecological studies of nonhuman species. The loss of phenotypic intraspecific variation—variation in foraging, diet and physiological strategies, social organization, and life‐history strategies—is potentially just as important to ecological extinction as the loss of entire species (Booke, 1981; Brown, Agee, & Franklin, 2004; Jesmer et al., 2018). Within *H. sapiens*, the hunter‐gatherer end of the life‐history strategy gradient, with its many regional and local variants, is currently threatened with biocultural extinction (Rapport & Maffi, 2010).

Up to 15 hominins (the clade more closely related to *Homo sapiens* than to chimpanzees and bonobos) evolved and went extinct between the Plio‐Pleistocene and the Holocene (Table 1; Wood, 2017; Wood & Lonergan, 2008; Wood & Richmond, 2000). We arbitrarily choose the Plio‐Pleistocene here as our starting point. All known extinct hominins were likely to have been group‐living omnivores, some of which coexisted, at least temporarily (Klein, 2005; Pickering, 2006). Although many snapshots of hominin species' diets in particular places and times are emerging (Blasco & Peris, 2012; Boschian & Saccà, 2015; Ferraro et al., 2013; Hardy & Moncel, 2011; Henry, Brooks, & Piperno, 2014; Macho, 2014), compared to living species, less is known about the ancient hominin niche and their phenotypes in the broad sense (Richards, 2002).

**Table 1:** Hominin species since the beginning of the Pleistocene, following Wood (2017)

Species	Category
*Homo sapiens*	Anatomically modern Homo
*H. neanderthalensis*	Premodern Homo
*H. heidelbergensis*	Premodern Homo
*Denisovans*	Premodern Homo
*H. naledi*	Premodern Homo
*H. rhodesiensis*	Premodern Homo
*H. antecessor*	Premodern Homo
*Dmanisi*	Premodern Homo
*H. ergaster*	Premodern Homo
*H. erectus*	Premodern Homo
*H. florsiensis*	Premodern Homo
*H. habilis*	Transitional hominins
*H. rudolfensis*	Transitional hominins
*Australopithecus africanus*	Archaic hominins
*Au. sediba*	Archaic hominins
*Paranthropus robustus*	Megadont and hyper‐megadont archaic hominids
*Au. garhi*	Megadont and hyper‐megadont archaic hominids
*P. boisei*	Megadont and hyper‐megadont archaic hominids
*P. aethiopicus*	Megadont and hyper‐megadont archaic hominids

The guild of large omnivores shares (by definition) some niche and phenotype features with *H. sapiens*, including flexible and generalist feeding strategies, and a suite of disturbance and ecosystem engineering/niche construction behaviors, though at lower intensities and complexities than found in *H. sapiens*. We discuss specific examples of these below. Similarly to *H. sapiens*, other large omnivores are opportunistic foragers, targeting areas with nonmobile prey and hunting for mobile prey as they encounter it (Bastille‐Rousseau, Fortin, Dussault, Courtois, & Ouellet, 2011; Kelly, 1995). There does not seem to be any literature on sex‐based specialization in foraging strategies in large omnivores other than *H. sapiens*. We are also not aware of studies on intraspecific variation in phenotypic strategies in large omnivores, though this may be a lacuna in the data rather than a unique feature of *H. sapiens*.

Omnivores have a high proportion of historical extinction and contemporary threat, with one third (32%) of terrestrial Pleistocene/Holocene large omnivores extinct, and of those remaining, half (51%) classed as at least “vulnerable to extinction” (Table 2). Large omnivores are not present in all ecosystems, but they are widespread (Terradas & Penuelas, 2011; Thompson, Hemberg, Starzomski, & Shurin, 2007). Despite considerable attention and debate about the outcomes of top predator loss and megaherbivore defaunation (Ripple et al., 2014, 2015), there seem to be few equivalent field studies of the effects of defaunation on large omnivore loss, or the effects of large omnivore loss on ecological functioning.

**Table 2:** This table combines entries from PANtheria (Jones et al., 2009) and from MammalDIET (Kissling et al., 2014)

Order	Family	Genus	Species	Terrestrial	Freshwater	Marine	Flying	Size (g)	Trophic Level	Mass (kg)	Diet	Density	Status
Primates	Hominidae	Homo	habilis							44			**X**
Primates	Hominidae	Pan	troglodytes							45	6	1.27	**E**
Cetartiodactyla	Suidae	Sus	philippensis	1	0	0	0	44,253.5	2				**V**
Cetartiodactyla	Suidae	Sus	oliveri	1	0	0	0	44,801.2	2				**V**
Cingulata	Dasypodidae	Dasypus	bellus	1	0	0	0	45,000	2				**X**
Carnivora	Otariidae	Arctocephalus	australis	1	0	1	0	45,000	2				LC
Carnivora	Otariidae	Callorhinus	ursinus	1	0	1	0	45,133	2				**V**
Cetartiodactyla	Suidae	Potamochoerus	larvatus	1	0	0	0	48,781.3	2	69.063	7	18	LC
Primates	Hominidae	Homo	sapiens	1	0	0	0	53,000	2	75	7		LC
Cetartiodactyla	Suidae	Sus	celebensis	1	0	0	0	55,000	2	53.813	5	0.23	NT
Cetartiodactyla	Suidae	Sus	ahoenobarbus	1	0	0	0	56,749.7	2				NT
Primates	Hominidae	Pongo	abelii	1	0	0	0	56,750	2				**CR**
Primates	Hominidae	Homo	erectus	1	0	0	0	57,000	2	60			**X**
Primates	Hominidae	Pongo	pygmaeus	1	0	0	0	57,150	2	53.408	5		**E**
Cetartiodactyla	Suidae	Potamochoerus	porcus	1	0	0	0	70,000.3	2	70	5	0.5	LC
Cetartiodactyla	Suidae	Sus	barbatus	1	0	0	0	70,500	2	135.805	6		**V**
Cetartiodactyla	Bovidae	Cephalophus	silvicultor	1	0	0	0	72,500.3	2	62.006	5	1.63	LC
Carnivora	Ursidae	Arctotherium	wingei	1	0	0	0	73,498.6	2				**X**
Cetartiodactyla	Tayassuidae	Mylohyus	nasutus	1	0	0	0	75,000	2				**X**
Primates	Hominidae	Homo	Denisovans	1	0	0	0	76,000	2				**X**
Primates	Hominidae	Homo	neanderthalensis	1	0	0	0	76,000	2	62.5			**X**
Carnivora	Otariidae	Zalophus	californianus	1	0	1	0	80,000	2	137.194	6		LC
Carnivora	Otariidae	Zalophus	japonicus	1	0	1	0	80,000	2				**X**
Carnivora	Otariidae	Zalophus	wollebaeki	1	0	1	0	80,000	2				**EN**
Carnivora	Otariidae	Arctocephalus	tropicalis	1	0	1	0	84,000	2	92.222	6		LC
Cetartiodactyla	Suidae	Babyrousa	celebensis	1	1	0	0	84,327.5	2				**VU**
Primates	Hominidae	Homo	heidelbergensis							90			**X**
Primates	Hominidae	Homo	antecessor							90			**X**
Carnivora	Phocidae	Histriophoca	fasciata	1	0	1	0	90,000	2	90	6		DD
Carnivora	Phocidae	Pusa	hispida	1	1	1	0	90,900	2	70.963	6	0.01	LC
Cetartiodactyla	Suidae	Sus	verrucosus	1	0	0	0	92,500	2	89.406	3		**E**
Carnivora	Ursidae	Melursus	ursinus	1	0	0	0	93,130	2	99.999	6		**V**
Carnivora	Otariidae	Arctocephalus	philippii	1	0	1	0	95,000	2	94.999	6		NT
Cetartiodactyla	Suidae	Babyrousa	babyrussa	1	1	0	0	100,000	2	92.95	6		**V**
Cetartiodactyla	Suidae	Sus	bucculentus	1	0	0	0	101,052.1	2				**X?**
Cetartiodactyla	Suidae	Sus	scrofa	1	0	0	0	101,052.1	2	84.471	5	3.54	LC
Carnivora	Otariidae	Arctocephalus	forsteri	1	0	1	0	101,250	2	101.249	6		LC
Cetartiodactyla	Tayassuidae	Platygonus	compressus	1	0	0	0	110,000	2				**X**
Carnivora	Ursidae	Arctotherium	tarijense	1	0	0	0	110,170	2				**X**
Carnivora	Otariidae	Phocarctos	hookeri	1	0	1	0	112,300	2	273.499	7	0.09	**V**
Cetartiodactyla	Suidae	Babyrousa	togeanensis	1	1	0	0	113,762.8	2				**V**
Carnivora	Phocidae	Pagophilus	groenlandicus	1	0	1	0	120,000	2				LC
Primates	Hominidae	Gorilla	gorilla	1	0	0	0	120,950	2	112.588	3		**CE**
Primates	Hominidae	Gorilla	beringei	1	0	0	0	130,000	2				**CE**
Carnivora	Otariidae	Otaria	flavescens	1	0	1	0	140,000	2	193.67	6		LC
Carnivora	Ursidae	Tremarctos	ornatus	1	0	0	0	140,000.6	2	123.176	5		**V**
Artiodactyla	Suidae	Metridiochoerus	compactus							142			**X**
Pilosa	Megalonychidae	Megalocnus	rodens	1	0	0	0	149,968.5	2				**X**
Carnivora	Phocidae	Phoca	largha	1	0	1	0	150,000	2	98.879	6	4.82E−03	DD
Carnivora	Ursidae	Tremarctos	floridanus	1	0	0	0	150,000	2	150			**X**
Carnivora	Ursidae	Ursus	arctos	1	0	0	0	180,520.4	2	196.287	6	0.11	LC
Artiodactyla	Suidae	Sus	cebifrons							190.792	6	0.4	**CE**
Artiodactyla	Suidae	Sus	philippensis							190.792	6	0.95	**V**
Cetartiodactyla	Hippopotamidae	Phanourios	minutes	1	0	0	0	200,000	2				**X**
Perissodactyla	Tapiridae	Tapirus	terrestris	1	1	0	0	207,500.9	2	169.496	5		**V**
Carnivora	Phocidae	Monachus	schauinslandi	1	0	1	0	223,000	2	222.999	6		**CE**
Carnivora	Phocidae	Monachus	monachus	1	0	1	0	275,000	2	294.881	6	0.02	**CE**
Carnivora	Phocidae	Erignathus	barbatus	1	0	1	0	280,000	2	279.999	6	0.85	LC
Carnivora	Phocidae	Cystophora	cristata	1	0	1	0	288,333.5	2	278.896	6	0.5	**V**
Carnivora	Ursidae	Arctodus	pristinus							300			**X**
Cetartiodactyla	Hippopotamidae	Hexaprotodon	sivalensis	1	1	0	0	300,000	2				**X**
Carnivora	Otariidae	Eumetopias	jubatus	1	0	1	0	310,000	2	382.466	6		NT
Carnivora	Phocidae	Hydrurga	leptonyx	1	0	1	0	360,000	2	352.675	6		LC
Carnivora	Phocidae	Leptonychotes	weddellii	1	0	1	0	360,000	2	400	6		LC
Carnivora	Ursidae	Ursus	maritimus	1	0	1	0	388,750.4	2	371.703	3	7.05E−03	**V**
Cetartiodactyla	Hippopotamidae	Hippopotamus	lemerlei	1	1	0	0	500,034.5	2				**X**
Cetartiodactyla	Hippopotamidae	Hippopotamus	madagascariensis	1	1	0	0	500,034.5	2				**X**
Carnivora	Ursidae	Arctodus	bonariensis							600			**X**
Carnivora	Ursidae	Arctodus	simus							720			**X**
Carnivora	Phocidae	Mirounga	angustirostris	1	0	1	0	750,000	2	1,112.39	6		LC
Carnivora	Odobenidae	Odobenus	rosmarus	1	0	1	0	825,000	2				**V**
Cetartiodactyla	Hippopotamidae	Hippopotamus	amphibius	1	1	1	0	1,417,490	2	1536.31	2	2	**V**

Note

Omnivores are listed in order of increasing mass. The columns “Terrestrial,” “Marine,” “Freshwater,” “Flying,” “Size,” and “Trophic level” come from MammalDIET, while “Mass,” “Diet,” and “Density” come from PANtheria. While 2 indicates omnivory in the trophic level classification of MammalDIET, PANtheria lists the number of trophic levels eaten from under “Diet.” “Status” refers to IUCN extinction risk status. Extinct species are shaded. Some animals that are typically considered not be omnivores are included here, such as hippopotamus; our table simply reflects the source databases.

We thus identify a set of interrelated underdeveloped areas in the literature on ecological extinction and defaunation. Next, we briefly consider evidence for the kinds of ecological functions and processes that may be missing: those of the omnivore guild where it experiences defaunation, of the Homo genus, and of the *H. sapiens* hunter‐gatherer phenotype where it has been or is being lost. The historical presence of different *H. sapiens *phenotypes in the broad sense, hominins, and other large omnivore species varies with location and is not equivalent in all areas (Faurby & Svenning, 2015; Sandom, Faurby, Sandel, & Svenning, 2014). We argue that especially where they existed on longer time scales, their loss may have had ecological consequences that have been inadequately considered.

## 4 THE NICHES AND FUNCTIONAL ECOLOGY OF LARGE OMNIVORES, HOMININS, AND *HOMO SAPIENS*

Functional traits are phenotypic traits of organisms that enable and control the rates and distributions of ecosystem processes (Díaz et al., 2007). Functional ecology is the study of the links between species phenotypes and ecological processes. Research on animal functional traits is somewhat underdeveloped (Hortal et al., 2015); for example, there are no authoritative lists of animal functional traits and their relationship to ecosystem processes comparable to those existing for plants. However, animal functional traits are extremely interesting due to the many ecosystem processes that animals affect, through trophic and nontrophic interactions such as creating intermediate disturbances, herbivory, predation and associated cascading effects, or ecosystem engineering and niche construction (Vanni, 2002; Wall & Moore, 1999). Understanding the key functional traits and resulting ecological functions and spatiotemporal patterns that are lost to defaunation or extinction is thus critical to selecting the most ecologically equivalent proxy species, when the lost species itself cannot be reintroduced (Chalcraft & Resetarits, 2003; Searcy et al., 2016).

We first consider the ecological functions of omnivores, to provide a basis for ecological comparisons between the omnivore guild in general, and specific functions of *Homo sapiens* and the extinct species of the Homo genus.

**Figure 2:**
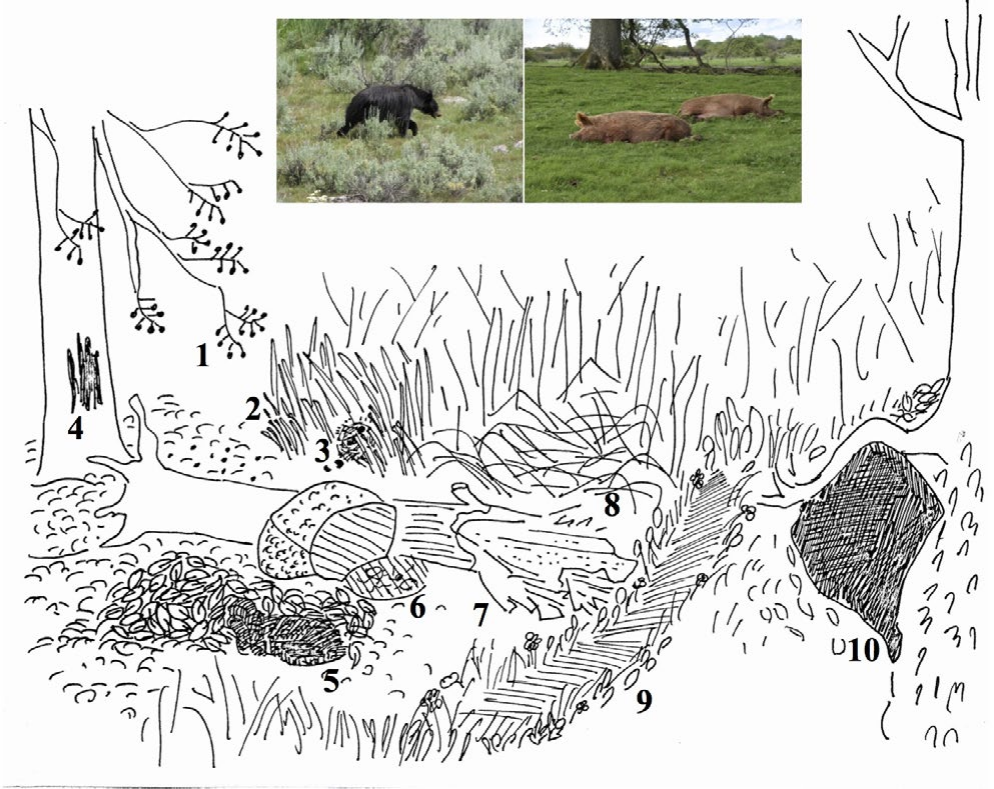
Some generic ecosystem impacts of large omnivores. Large omnivores eat, for example, (1) berries, seeds, and nuts, (2) grasses and other plants, (3) eggs. (4) They damage tree bark, (5) dig in soil and litter, (6) overturn rocks, and (7) help break down rotting wood while foraging for invertebrates and other food sources. They (8) make nests, (9) trails, and (10) burrows. They also connect, as shown, a variety of habitat types. Image © MR‐B. Inset: Examples of large omnivores: black bear *Ursus americanus* and semi‐wild Tamworth pigs (photographs © Jens‐Christian Svenning)

Omnivores appear to have some distinctive ecological functions (Figure 2). Omnivores often maintain multiple weak links across trophic levels, which contribute to long‐term persistence and resilience of food webs (Gellner & McCann, 2011; Kratina et al., 2012; Stouffer & Bascompte, 2010). Due to their ability to exploit spatially and temporally discrete resources, omnivores can damp resource pulses at both the primary and secondary trophic levels (Shaner & Macko, 2011; Visser, Mariani, & Pigolotti, 2012). For illustrative and comparative purposes, here we provide some specific examples of the ecological roles of selected large omnivores. Bears eat grasses, forbs, ferns, fruits, roots and tubers, insects, honey, birds, ungulates (particularly neonates), and mammal carrion, in different proportions across the year, and across habitats (Laurie & Seidensticker, 1977; Munro, Nielsen, Price, Stenhouse, & Boyce, 2006; Noyce, Kannowski, & Riggs, 1997; Servheen, 1983). Bears predate large as well as a range of medium‐sized and small prey species (Stirling & Derocher, 1990). Pigs also eat fruits, seeds, invertebrates, eggs, reptiles, birds and mammals, carrion, roots, and grasses (Ghiglieri, Butynski, & Struhsaker, 1982; Leus & MacDonald, 1997; Skinner, Breytenbach, & Maberly, 1976). These represent some of the species, taxonomic groups, functional traits, or nutrient pools that these large omnivores may regulate. Omnivores also provide a range of ecological disturbances. While foraging, large omnivores may dig in the soil, overturn rocks, and help to break down fallen trees, affecting soil processes for example (Andriuzzi & Wall, 2018; Ghiglieri et al., 1982; Laurie & Seidensticker, 1977; Munro et al., 2006; Skinner et al., 1976). *Sus scrofa* is considered an ecosystem engineer due to the impacts of its extensive disturbances created during foraging (Barrios‐Garcia & Ballari, 2012). Grizzly bear digging for lily bulbs has an ecosystem engineering effect increasing soil nitrogen and promoting the growth of lilies (Tardiff & Stanford, 1998). Black bears *Ursus americanus* and brown bears *Ursus arctos* in a wide range of habitats excavate dens, which provide an insulated microhabitat (LeCount, 1980; Miller, 1990). Grizzly bears and wild boar create nests from depressed vegetation (Barrios‐Garcia & Ballari, 2012; Munro et al., 2006) affecting microhabitat structure, the microclimate, and potentially the accumulation of plant litter. Bears and pigs both scratch trees (Heinken, Schmidt, Oheimb, Kriebitzsch, & Ellenberg, 2006; Laurie & Seidensticker, 1977; Skinner et al., 1976), damaging the bark and providing a niche for molds and fungi, which may contribute to tree death and forest succession (Schmidt, 2006). Pigs may make and use trails (Blouch, 1988), which can affect plant and animal biodiversity and seed and nutrient dispersal patterns. Trails could also act as natural fire breaks, while foraging by digging in leaf litter and soil can reduce fuel loads: These functions will affect fire intensity and distribution and thus successional dynamics (Johnson et al., 2018). Both bears and pigs act as seed dispersers (Ghiglieri et al., 1982; Heinken et al., 2006; McConkey & Galetti, 1999). Large omnivores are also often habitat generalists, or inhabit habitat mosaics, and thus could be implicated in landscape mosaic dynamics (Munro et al., 2006; Saunders & Giles, 1995; Servheen, 1983). Large omnivores could also be important long‐distance link species (Lundberg & Moberg, 2003); salmon‐eating brown bears, for example, are a key link moving oceanic nutrients into terrestrial forests (Gende, Miller, & Hood, 2007; Holtgrieve, Schindler, & Jewett, 2009). Brown bears are adapted to a wide range of environmental conditions and thus may create ecological connections between distinct habitats through foraging and dispersal (Fergusen & McLoughlin, 2000). Wild boars flexibly adjust their home ranges depending on ecological conditions (Morelle et al., 2014). While bears tend to be solitary, pigs often form fission–fusion social structures (Ghiglieri et al., 1982). These social structures and territorial patterns will lead to different spatial distributions of ecological functions and processes.

Hominins, as large omnivores, almost certainly had similar ecological functions and characteristics. For example, they certainly varied in their trophic level, with Neanderthals obtaining most of their protein from larger herbivores, while comparable *Homo sapiens* populations in Europe ate a wider variety of smaller herbivores and fish (Richards & Trinkaus, 2009). Hominin trophic levels and diets also changed with location and time (Weyrich et al., 2017). During the Pleistocene, *Homo* spp. evolved a hunter‐gatherer strategy concurrent with the evolution of cognitive and social adaptations for group hunting, meat and plant part processing, and food storage (Ungar, Grine, & Teaford, 2006). Gatherable, slow‐maturing, overexploitable species such as tortoises and shellfish were supplemented, by the Pleistocene–Holocene transition, with hunting and trapping of fast‐moving small game with rapid population growth, such as lagomorphs and small birds (Stiner, 2002). While bears hibernate and wild pigs dig for tubers to survive winter food shortages, *Homo *spp. in temperate climates may have primarily relied on hunting high‐fat adult ungulates, a foraging tactic in some cases dominated by males as in contemporary *Homo sapiens* hunter‐gatherers (Stiner, 2002). Kuhn et al. (2006) argue that this sex‐based specialization in foraging emerged only around 50,000 years ago. Prior to this, hominins, including Neanderthals, appear to have lived in highly cooperative groups without clear specializations in foraging. The male specialization for hunting large prey, nevertheless, developed before the Late Pleistocene/early Holocene megafaunal extinctions in which it may have played a critical role (Sandom et al., 2014). Kuhn et al. (2006) also emphasize that even within modern *H. sapiens* societies with sex‐based foraging specializations, foraging strategies are flexible, and roles are exchangeable between sexes under various circumstances, and may change over the lifetime of an individual. This intraspecific and indeed intra‐individual variation is important to keep in mind and suggests that *Homo* spp. foraging strategies are not, on the whole, significantly different from those of other large omnivores. We can thus assume that the now‐extinct hominins had comparable ecological functions and affected ecological processes in ways similar to those discussed above, for other omnivores.

*Homo sapiens* hunter‐gatherers have roles in a wide range of ecosystem processes. Since a comprehensive review of *H. sapiens* hunter‐gatherer ecological roles would include the entire fields of ethnography, human ecology, and archaeology, we do not claim to be exhaustive. However, it should be noted that not only do hunter‐gatherers have many different kinds of ecological roles, there is a strong functional trait x environment interaction (Kelly, 1995), and also variation among the outcomes of all these different interactions, ranging from very positive to neutral to very negative outcomes, at various scales (see also next section). The hunter‐gatherer phenotype (as well as along the gradient toward agricultural strategies) has many commensal and mutualistic species, which benefit from *H. sapiens* disturbances and niche construction (Diamond, 2002; Keller, 2007; Lundholm & Richardson, 2010). For some plants, *H. sapiens* appears to have emerged as a substitute mutualistic partner after the extinction of megafauna (Kistler et al., 2015). *Homo sapiens* gathering can create trophic cascades that favor increased biodiversity (Castilla, 1999). Comberti, Thornton, Echeverria, and Patternson (2015) have described as “services to ecosystems” a suite of ecological roles performed by *H. sapiens* in the Amazon*, *including coevolution, facilitation, seed dispersal, niche construction of aquatic or inundation‐free sites, burning, soil improvement, and habitat creation for other species. Although there is more research on the ecological roles of *H. sapiens* than for the extinct species of the Homo genus or for the omnivore guild, this area of research is still patchy and not well integrated with standard ecological approaches.

In Table 3, we have attempted to summarize some key ecological processes contributed to by *H. sapiens*, and the functional traits of *H. sapiens* that support them. We approached the task of summarizing *H. sapiens* hunter‐gatherer ecological functions and functional traits comparatively, by consulting papers on nonhuman animal functional traits, and indicatively, drawing on the literature on *H. sapiens* hunter‐gatherers. While there is no morphological difference underlying the different ecological functions across agriculturalist and hunter‐gatherer strategies, their functional traits nonetheless interact with the environment differently, given their phenotypes in the broad sense; we did not attempt to summarize agriculturalist ecological roles, which are not as a whole endangered (though of course many agricultural practices are in decline, such as transhumance; we do not wish to minimize the importance of the loss of traditional small‐scale agricultural practice diversity, but it is, perhaps arbitrarily, outside the scope of this paper). We indicate functional traits for seed dispersal, soil formation and disturbance, interactions with fire, changes to the hydrological cycle and the nutrient cycle, population control of various taxa, and mutualisms affecting the ecological functions of other species. We can recognize at least three major kinds of ecological roles of *H. sapiens* that can have positive (biodiversity increasing) outcomes: niche construction/coevolution, facilitation/mutualism, and disturbance/predation.

**Table 3:** Some functional traits of hunter‐gatherer *Homo sapiens* and their relations to some ecological processes

Trait	Associated process	Type of process	Suggested measure	Indicative specific examples or review papers
Fruit‐eating	Seed dispersal via endozoochory	Facilitation/mutualism	Gape width Incisor length Fruit‐opening technology Tree climbing height	Ungar (1996), Pires et al. (2014), and Kraft, Venkataraman, and Dominy (2014)
Basket technology			Volume of basket relative to fruit, fruit load Home range	Oswalt (1972)
Defecation microhabitat			Overlap with germination microhabitat	Bassotti and Villanacci (2013) and Reinhard, Hevly, and Anderson (1987)
Hairiness	Seed dispersal via ectozoochory (epi‐anthropochory)	Facilitation/mutualism	Height Hair density Hair length	Rantala (2010)
Clothing			Clothing material (adhesiveness) Clothing area Clothing height	Wichmann et al. (2008), Ansong and Pickering (2014)
Bee keeping	Pollination mutualisms	Facilitation/mutualism	Bee population Bee‐pollinated plant populations	Dale and Ashley (2010)
Fire technology	Fire regulation: area affected and intensity	Disturbance/predation	Type of technology Home range (dispersal) Frequency of use	Roebroeks and Villa (2011), Archibald, Staver, and Levin (2012)
Trail formation			Area, frequency, density	Johnson et al. (2018)
Digging in soil and litter			Area, frequency, density	
*Terra preta* or trash heaps	Soil formation	Niche construction/coevolution	Rate of accumulation Volume formed	McMichael et al. (2012) and Schmidt (2013)
Dams, canals, drainage	Hydrological cycling	Niche construction/coevolution	Associated practices and technologies	Williams et al. (2014)
Defecation microhabitat	Nutrient flux	Niche construction/coevolution	Habitat distribution of each	Foster (1999), Andriuzzi and Wall (2018)
Burial microhabitat				Brandt (1988)
Trampling	Bioperturbation	Disturbance/predation	Area, frequency, density	Ejrnæs (2015), Mason et al. (2015), Root‐Bernstein and Svenning (2018)
Trail formation			Area, frequency, density	
Digging in soil and litter			Area, frequency, density, digging tool technology	
Hunting and gathering	Herbivore (bird, mammal, reptile, fish, mollusk, etc.) population control	Disturbance/predation	Hunting technology, species richness hunted, rate of kill Body size Running speed, endurance, limb length Diet Tooth morphology	Oswalt (1972), Kelly (1995) and Lieberman and Bramble (2007)
Hunting and gathering	Carnivore (bird, mammal, fish, etc.) population control	Disturbance/predation	Hunting technology, species richness hunted, rate of kill Body size Running speed, limb length Diet Tooth morphology	Oswalt (1972) and Kelly (1995)
Hunting and gathering	Arthropod control	Disturbance/predation	Diet	Oswalt (1972) and Kelly (1995)
Scavenging	Disease and parasite lifecycle control	Niche construction/coevolution	Frequency Time to clean carcass Volume consumed Tooth/tool morphology	Lieberman and Bramble (2007) and Pickering and Bunn (2007)
Defecation microhabitat			Distance to food preparation, gathering Distance to water	Bassotti and Villanacci (2013) and Reinhard et al. (1987)
Medicinal ethnobotany			Number of species uses known	Elanchezhian, Kumar, Beena, and Suryanarayana (2007)
Planting	Plant community succession	Facilitation/mutualism	Species richness planted	Michon, De Foresta, Levang, and Verdeaux (2007), Manner (1981) and Zvelebil and Rowley‐Conwy (1984)
Weeding			Species richness weeded	
Clearing (swidden)			Frequency, area, density Tool use	
Association with “beater” birds or honeyguides	Feeding mutualisms or other mutualisms	Facilitation/mutualism	Frequency	Whelan, Wenny, and Marquis (2008)
Domestication			Frequency Abundance	Larson et al. (2012)

Note

Some “traits” that *Homo sapiens* make or build are also included (cf. the “extended phenotype” or “constructed niche”). Quantitative measures are suggested wherever possible. “Control” may refer either to increase or decrease. The “indicative specific examples” column contains at least one example of a paper primarily from the ecology literature or a closely related literature, attending to this set of traits and/or the ecological processes it contributes to. There are obviously hundreds if not thousands of ethnographic examples for each category but it was beyond our capacity to cite all of these. For the types of processes, “niche construction/coevolution” refers to interactions linking biotic and abiotic processes, and feeding back to evolution; “facilitation/mutualism” refers to interactions that allow or increase ecological processes, and “disturbance/predation” refers to ecological processes characterized by destruction/conversion of biomass.

By contrast, the widespread, negative ecological impacts of the agriculturalist phenotype cluster, or specifically, the variant that is currently most abundant (“conventional agriculture”), are well known and abundantly researched. Negative impacts include reduction in coevolved ecological interactions, reduction in degree of *H. sapiens* omnivory, increases in environmental disturbances leading to degradation, and reduction in the frequency and extent of other agriculturalist strategies (at different points along the gradient) with lower risks of degradation impacts. Many of the existing ecological interactions within agricultural strategies, some of them involving coadaptations between species, are currently at risk of ecological extinction, leading to loss of agrobiodiversity (Gepts et al., 2012). Within *H. sapiens*, the emergence of agriculturalist populations and the increasing dominance of agriculture as a life‐history strategy had the effect of exposing *H. sapiens* to acute and chronic malnutrition, resulting in a low mean trophic level, equivalent to a reduction in degree of omnivory (Benyshek & Watson, 2006; Pearson, 1997). The trophic level is now rising globally with the expansion and continued development of the agriculturalist *H. sapiens* constructed niche, notably the emergence of the so‐called “conventional agriculture” strategy, which however comes at the cost of a regime of severe chronic and pulse disturbances and environmental degradation (Bonhommeau et al., 2013; Darimont, Fox, Bryan, & Reimchen, 2015; Pearson, 1997; Richards, 2002).

Various approaches to estimating the impact of *H. sapiens* on the global environment have been proposed (Ellis et al., 2013). A new era, the Anthropocene, has been proposed to indicate the period of *H. sapiens'* planetary impact, starting around 1945, but with important antecedents since the emergence of the *Homo* genus (Crutzen, 2002; Foley et al., 2013). These impacts are largely attributable to the more extreme end of the agriculturalist phenotype gradient, although significant debates are ongoing about the contribution and roles of other phenotype clusters along this gradient. While there is a tendency to view this as an irreversible and accelerating ecological/evolutionary transition toward unsustainable environmental destruction, various scholars have pointed out that variations in *H. sapiens* behaviors with different ecological impacts are still possible (Roelvink, St. Martin, & Gibson‐Graham, 2015; Svizzero & Tisdell, 2015).

## 5 NEGATIVE AND POSITIVE IMPACTS OF REINTRODUCING SOME *H. SAPIENS* ECOLOGICAL FUNCTIONS

Restoration of the ecosystem functions discussed above, where they are missing, is broadly expected to have positive ecosystem impacts, although as with any restoration, reintroduction, or rewilding context, the scale and habitat specificity of the reinstated trophic and nontrophic interactions need to be carefully considered to ensure that negative impacts are avoided or minimized. The native status or temporal depth of large omnivore, hominin, and hunter‐gatherer *H. sapiens* populations in different areas will play a part in determining the expected historical and future impacts of their ecological roles (Faurby & Svenning, 2015; Sandom et al., 2014). In many cases, restoration of ecosystem functions may be possible through improved species‐ and population‐focused conservation actions or reintroductions. However, in the remainder of the paper we concentrate on another possibility that we think is promising where such traditional approaches fail or are not feasible, that of *H. sapiens* acting as a substitute for lost ecological functions.

Research on proxy species and taxon substitutes for various restoration scenarios to reverse defaunation, as discussed in the Section 2, has considered a wide range of candidate taxa, notably tortoises (Griffiths, Zuel, Jones, Ahamud, & Harris, 2013) and mammalian large fauna including horses and cattle (Naundrup & Svenning, 2015; Navarro & Pereira, 2015; Taylor, 2009; Sandom, Hughes, & Macdonald, 2012; Wilder et al., 2014). This approach has been criticized with the argument that proxy species introductions, like invasive species introductions, could reduce biodiversity, damage ecosystem and evolutionary processes, and put *H. sapiens* populations at risk (Nogues‐Bravo, Simberloff, Rahbek, & Sanders, 2016; Rubenstein, Rubenstein, D, Sherman, & Gavin, 2006). There is no standard definition of invasive species, but generally they are considered to be species that are outside their native range, have a rapidly increasing population or have become widespread and abundant, and harm the ecosystem (Colautti & MacIsaac, 2004). This is particularly relevant for *H. sapiens* as a potential proxy species. Many ecologists are accustomed to consider only the negative ecological impacts of *H. sapiens* and will undoubtedly be skeptical that it can safely be used as a taxon substitute. Researchers in conservation, restoration, and rewilding commonly use significant *H. sapiens* impact as a baseline, and seek to restore to before that impact in many cases (Corlett, 2013; Jachowski, Kesler, Steen, & Walters, 2015; Svenning et al., 2015). Here, we consider whether *H. sapiens* is an invasive species, and whether its negative environmental impacts can be decoupled from its positive ecosystem functions. Essentially, this is another version of an argument about whether *H. sapiens* has significant intraspecific variation in its ecological impacts, and/or whether the most environmentally destructive forms of agriculturalist strategy are on an irreversible path to dominating the planet and the species (whether “things can be different because they already are,” or not).

The effects of *H. sapiens*' activities on species and landscapes are widespread, profound, and historically old (Ellis et al., 2013). However, outside Africa and southern Eurasia, these impacts are recent in an evolutionary sense (≤50,000 years in Australia; ≤15,000 years in the Americas, and <1,000 years on New Zealand and many other islands; Sandom et al., 2014). Some definitions of invasiveness include the characteristic that the species range expansion is facilitated by *H. sapiens *(see e.g., Lee, 2002). By this definition, the original and all subsequent range expansions of *H. sapiens* from Africa were cases of invasion by self‐facilitation. In addition, there are undoubtedly negative impacts of *H. sapiens *range expansions. As noted above, the dominant varieties of agriculturalist strategy are now causing widespread negative environmental impacts (often with a considerable lag time after the original range expansion and/or due to secondary range expansions) (Bonhommeau et al., 2013; Darimont et al., 2015; Pearson, 1997; Richards, 2002). But the hunter‐gatherer phenotype can also have negative ecosystem impacts. Populations of hunter‐gatherer *H. sapiens* have been implicated in the extinction of many other species, including megafauna, island species, and others (Grayson, 2001; Holdaway et al., 2014; Kay, 1994; Koch & Barnosky, 2006; Martin, 1984; Martin & Szuter, 1999; Sandom et al., 2014). The small‐scale disturbances of hunter‐gatherer *H. sapiens* may locally minimize biodiversity at certain spatiotemporal scales (Anthony, Marriner, & Morhange, 2014; Barlow, Gardner, Lees, Parry, & Peres, 2012; Bishop, Church, & Rowley‐Conwy, 2015; Feurdean et al., 2012; Kuneš, Pokorný, & Šída, 2008; Muler et al., 2014). *Homo sapiens'* hunting and gathering of the largest individuals within species reduces the value of large size as a defense structure and has been suggested to reduce the genetic variation needed to evolve new defense strategies (Vermeij, 2012). These negative impacts can be enough to establish the invasive status of *H. sapiens* of both phenotypes throughout its range, with the sole exception of some African populations (which, without being invasive, may still have negative impacts). This constitutes a serious argument against using *H. sapiens* as a taxon substitute (compare Nogues‐Bravo et al., 2013), if we assume that these negative impacts are the result of invariant and fundamental functional traits of *H. sapiens*.

However, an appealing aspect of using *H. sapiens* as a taxon substitute is the possibility to draw selectively on its functional traits, taking advantage of its learning capacities and phenotypic flexibility. *H. sapiens* obviously cannot learn to perform any ecological function that exists, but of those in Table 3, individuals may be able to learn many variants. The examples of ecological functions in Table 3 in many cases have positive impacts on environmental variables (see also Angelsen et al., 2014; Barrett, Lee, & McPeak, 2005; Bauch, Sills, & Pattanayak, 2014; Belcher, Ruíz‐Pérez, & Achdiawan, 2005; Milder, Hart, Dobie, Minai, & Zaleski, 2014; Root‐Bernstein & Svenning, 2018; Sunderlin et al., 2005; Wunder, Angelsen, & Belcher, 2014). Projects can facilitate trait–environment interactions of *H. sapiens* that do not tend toward degradation and species extinctions.

Of the three main kinds of ecological functions summarized in Table 3, disturbance/predation (removal of biomass) is perhaps the kind of ecological role most likely to be expected to have negative impacts. However, the intermediate disturbance hypothesis states that high intensities reduce biomass, productivity, and species richness, but medium intensities increase it (Shea, Roxburgh, & Rauscher, 2004). This seems to be generally the case across forms of biomass destruction that could be described as either disturbance or predation, whether in terms of gathering mollusks, grazing herbaceous plants, or destruction through fire or trampling (Root‐Bernstein, 2013). Intensity of disturbance is notoriously difficult to measure (Shea et al., 2004). However, we estimate that medium intensities of disturbance may occur all across the gradient of hunter‐gatherer to agricultural strategies, although the impacts of those intensities should vary with environmental conditions and the ecological context. Bleige Bird (2015) argues that landscape‐scale intermediate disturbance may drive positive ecological feedbacks and may also lead to shifts along the phenotype strategy gradient. This perspective provides an argument for case‐by‐case analysis of the net environmental impacts of habitat‐specific *H. sapiens* phenotypes. As we describe in the next section, with examples, given existing documentation of positive effects of *H. sapiens* ecological roles, we believe it is possible for *H. sapiens* to learn to perform specific ecosystem functions at adjustable and appropriate rates and densities (Armitage et al., 2009; Cundill, Cumming, Biggs, & Fabricius, 2011; Folk, Hahn, Olsson, & Norberg, 2005).

The history of domination by agriculturalist *H. sapiens* populations and the decline or disappearance of hunter‐gatherers, other hominins and many large omnivores, may have contributed to a moving baseline effect that has blinded ecologists to the ecological effects of their functional extinctions (Anderson, Kelly, Ladley, Molloy, & Terry, 2011; Papworth, Rist, Coad, & Milner‐Gulland, 2009). Further research on the spatiotemporal patterns resulting from the particular foraging tactics, trophic interactions, and disturbance regimes of extant and extinct large omnivores (including *Homo* spp.) and *H. sapiens* hunter‐gatherers would help to develop more specific predictions about the possible impacts of their restoration where they are missing.

Finally, we provide a broad overview of approaches to using *H. sapiens* as a taxon substitute and the practical forms they might take. An “adaptive” approach assumes that a project has identified a contextual variation on the *H. sapiens* hunter‐gatherer phenotype, or a specific set of functional roles, that once had, or are expected to have, a net positive environmental impact. Secondly, the “proxy” approach is a use of specific *H. sapiens* behaviors and capabilities to generate a functional proxy for one of the many extinct or endangered large omnivores, including extinct hominins, in regions where they have a deep history.

We emphasize that, in our view, the appropriate way to employ *H. sapiens* as a taxon substitute is to facilitate, with free prior informed consent and respecting human rights (Boyle & Anderson, 1996; UNESCO, 2010), the spontaneous emergence of a self‐organizing sustainably functioning ecological system not requiring coercive management interventions by conservation managers or other outside actors. Self‐determination, and the integrity of culturally acquired behaviors and information are key considerations (UNESCO, 2018). However, we do not mean that populations of *H. sapiens* must each reinvent these ideas for themselves. We believe there is a clear role for facilitation through training, capacity building, and adaptive learning, if there is free prior informed consent for the exchange of ideas and practices (Cundill et al., 2011; Worm & Paine, 2016). Training and teaching can be coercive and unjust even when intentions are good: It is thus a responsibility of any conservation biologists wishing to implement any of our suggestions here in any kind of community to work with social scientists or development/humanitarian/social work experts, along with community members, in the design of any form of facilitation, and to take into account how knowledge frames, values, and power are interrelated (Batterbury, 2018; Borrini, Kothari, Oviedo, & Oviedo, 2004; Infield, Entwistle, Anthem, Mugisha, & Phillips, 2018; Reid et al., 2016; Stripple & Bulkeley, 2015; UNESCO, 2018). Any programs implementing any of the ideas sketched out below should be based on best practice in noncoercive exchange of ideas with self‐determining communities.

*Homo sapiens* could be employed as a proxy for many of the species we have focused on here (Table 3). While preserving the populations of endangered large omnivores is clearly a priority in its own right, in some cases it will not always be feasible to reintroduce them across their historical ranges, and proxies may be considered. Although *H. sapiens* spends time in freshwater and marine habitats, it is especially well adapted to a wide range of terrestrial habitats where the greatest losses of mega‐omnivores have occurred; for this reason, we focus on terrestrial contexts. Equally, where hunter‐gatherer groups still exist, the priority should go toward safeguarding or improving their own capacity to carry out ecological functions.

We discuss three examples that could be implemented either through a “proxy” or an “adaptive” approach as outlined above, including foraging, transhumance, and hunting. Our discussion focuses on the ecological aspects of these roles rather than the governance, political, economic, environmental justice, or socio‐ecological issues, which are very important but have been extensively treated elsewhere.

Foraging of plant parts and mushrooms (and sedentary animals or life‐history stages, e.g., eggs) is an activity present throughout the ranges of *H. sapiens*, the hominins, and other mega‐omnivores, compatible with many baselines up to the present day.There are examples of foraging for plant parts or mushrooms leading to extinctions or species endangered status, but harvest for illegal globalized markets plays a role in all or most of these (Swarts & Dixon, 2009). Thus, we do not recommend encouraging foraging for products sold on to distant, unregulated, or black markets.

Ecological functions that *H. sapiens* can help to reintroduce through foraging include provision of intermediate disturbance (paths and trampling) promoting biodiversity and succession (Ejrnæs, 2015) and seed dispersal (Pires et al., 2014), as well as potential food web stabilization via generalist omnivory (see above). As always, these potential benefits to ecosystem processes are habitat and context dependent. In Australia, for example, native plants do not appear to recover from *Homo sapiens* trampling even after a year, with potentially negative habitat impacts (Mason, Newsome, Moore, & Admiraal, 2015).

Hunting remains a very common activity of *H. sapiens* whether or not its primary function is to supplement the diet. Hunting strategies adapt to ecological and environmental conditions (Alves, Mendonça, Confessor, Vieira, & Lopez, 2009; Berkes & Jolly, 2002; Byers & Broughton, 2004; Gell, 1996). Thus, the local ecological knowledge (LEK) of hunters is often important to developing scientific management plans (Tidemann & Gosler, 2012; Tori, McLeod, McKnight, Moorman, & Reid, 2002). On the other hand, unregulated and illegal hunting (poaching) is a scourge for many species and has led to many extinctions or expected extinctions (Dirzo et al., 2014). We would not advocate encouraging indiscriminate hunting of the largest prey species, a phenomenon that has led to defaunation and “ecological shrinkage” throughout the tropics (Abernethy, Coad, Taylor, Lee, & Maisels, 2013; Corlett, 2013; Hansen & Galetti, 2009). Darimont et al. (2015) show that all contemporary *H. sapiens* hunters combined (sport, market, and subsistence) disproportionately target adult animals, which can also skew prey populations. Darimont et al. (2015) recommend emulating other predators' hunting patterns as a form of management. Here, we add that emulation of omnivore hunting patterns could contribute to restoring the lost influences of extinct or threatened large omnivores.

Comobility of *H. sapiens* and large herbivores, which evolved into forms of domestication, pastoralism, and transhumance (Niven et al., 2012), is a possible proxy function representing a movement along the gradient away from agriculturalist life‐histories. Many relationships between *H. sapiens* and large herbivores lie somewhere between transhumance and hunting, such as with wild vicuñas (*Vicugna vicugna*) and guanacos (*Lama guanicoe*), which are captured every couple of years and shorn for their wool before being released, a practice of the Inca that has recently been revived for sustainable exploitation (Bonacic, Feber, & Macdonald, 2006; Montes, Carmanchahi, Rey, & Funes, 2006). Reindeer (*Rangifer tarandus*) provide another example of herded and exploited semi‐wild large herbivores, with a number of forms of interaction with *H. sapiens* (Ingold, 1980). Large herbivore‐*H. sapiens* comobility is increasingly under threat (Sayre, McAllister, Bestelmeyer, Moritz, & Turner, 2013). Long‐distance migrations of wild megaherbivores are also threatened by land use changes (Ito et al., 2013). Restoring large‐scale comobility could contribute to a number of ecosystem processes, such as seed dispersal (Poschlod & Bonn, 1998), and top‐down vegetation control. Krader (1955) argues that nomadic pastoralism constitutes a nearly closed symbiosis, with for example the dung of the ungulates being collected to use for fuel, rather than returning to the soil. Thus, one approach to “rewilding” pastoralism to move toward spontaneous comobility might include returning a proportion of dung, carcasses, and so on, to the local detritivores, so that less biomass production is monopolized and diverted by *H. sapiens* (Krausman et al., 2013).

## 6 CONCLUSION

Ecologists increasingly recognize that *H. sapiens* has always been deeply enmeshed in ecological interactions. Despite widespread concern over the increasing negative environmental impacts of agriculturalist *H. sapiens*, almost no attention has been paid to the extinction of other ecological roles that this uniquely flexible species once played. Here, we have briefly considered the range of threatened or extinct functional roles of hunter‐gatherers, extinct hominins, and large omnivores. Conservationists will notice that our practical suggestions for using *H. sapiens* as a taxon substitute to replace some of these functions align closely with many existing livelihoods, conservation practices, and emerging management approaches around the world. Our suggestions thus represent minor changes in practice, but major changes in perspective. Ignoring the potential roles of *H. sapiens* within restoration projects overlooks their ecological roles as omnivores, their many commensalisms, mutualisms, disturbances, and niche constructions that can favor other species, their abundance and widespread distribution, and their ability to learn. It also ignores that they have previously, over evolutionary time, taken over the ecological roles of extinct species and are thus capable of doing so in the future (Kistler et al., 2015). This does not mean, in our view, that *H. sapiens* could or should aim to simulate and replace all or any nonhuman species' roles (Cantrell, Martin, & Ellis, 2017): Our argument is based on phenotype/niche similarities within the large omnivore guild, and the possibility of otherwise irreversible ecological extinctions. Because *H. sapiens* is capable of high levels of population density, trophic control, competition, and disturbance, many ecologists see it as fundamentally unlike nonhuman species, and inherently damaging to the environment. Using our comparative frame, we have attempted to show that this is a limited and biased view. Future research on reducing *H. sapiens* environmental harms should attend to the conditions under which *H. sapiens*' negative environmental impacts can be successfully decoupled from important positive ecological roles.

We argue that *H. sapiens* is both a fully ecological subject, fully comparable to other species in its ecology, and an excellent example of intraspecies phenotypic variation and plasticity, influenced by a wide range of environmental, developmental, and learning and behavioral factors and processes. These should not be contradictory. A focus on the ecology of *H. sapiens* highlights the tension within ecology between variation and plasticity on the one hand, and global, conceptual patterns and evolutionary generalities on the other. This tension is not unique to *H. sapiens* ecology; it is increasingly evident as new observations of nongenetic, habitat‐specific forms of adaptation, and ecological response to environmental change are accumulated in various biological and ecological disciplines. Perhaps thoughtful attention to the ways in which intraspecific variation and plasticity in *H. sapiens* can translate into species‐level ecological generalities, but also to how to translate species‐level ecological generalities into habitat and community‐level particularities, would be a helpful meeting point for cross‐disciplinary research.

## CONFLICT OF INTEREST

None declared.

## AUTHOR CONTRIBUTIONS

MR‐B and RL conceived of the paper, MR‐B wrote the paper, and RL edited it.

## Data Availability

As this is a conceptual paper, there are no data to share beyond the reference list.

## References

[ece35049-bib-0001] Abernethy, K. A. , Coad, L. , Taylor, G. , Lee, M. E. , & Maisels, F. (2013). Extent and ecological consequences of hunting in Central African rainforests in the twenty‐first century. Philosophical Transactions of the Royal Society B: Biological Sciences, 368(1625), 20120303. 10.1098/rstb.2012.0303 PMC3720024 23878333

[ece35049-bib-0002] Allendorf, F. W. , & Hard, J. J. (2009). Human‐induced evolution caused by unnatural selection through harvest of wild animals. Proceedings of the National Academy of Sciences of the United States of America, 106, 9987–9994. 10.1073/pnas.0901069106 19528656 PMC2702803

[ece35049-bib-0003] Alves, R. R. , Mendonça, L. E. , Confessor, M. V. , Vieira, W. L. , & Lopez, L. C. (2009). Hunting strategies used in the semi‐arid region of northeastern Brazil. Journal of Ethnobiology and Ethnomedicine, 5(1), 12 10.1186/1746-4269-5-12 19386121 PMC2678999

[ece35049-bib-0004] Anderson, S. H. , Kelly, D. , Ladley, J. J. , Molloy, S. , & Terry, J. (2011). Cascading effects of bird functional extinction reduce pollination and plant density. Science, 331(6020), 1068–1071. 10.1126/science.1199092 21292938

[ece35049-bib-0005] Andriuzzi, W. S. , & Wall, D. H. (2018). Soil biological responses to, and feedbacks on, trophic rewilding. Philosophical Transactions of the Royal Society B: Biological Sciences, 373, 20170448 10.1098/rstb.2017.0448 PMC6231063 30348874

[ece35049-bib-0006] Angelsen, A. , Jagger, P. , Babigumira, R. , Belcher, B. , Hogarth, N. J. , Bauch, S. , … Wunder, S. (2014). Environmental income and rural livelihoods: A global‐comparative analysis. World Development, 64, S12–S28. 10.1016/j.worlddev.2014.03.006 PMC7220182 32405139

[ece35049-bib-0007] Ansong, M. , & Pickering, C. (2014). Weed seeds on clothing: A global review. Journal of Environmental Management, 144, 203–211. 24956465 10.1016/j.jenvman.2014.05.026

[ece35049-bib-0008] Anthony, E. J. , Marriner, N. , & Morhange, C. (2014). Human influence and the changing geomorphology of Mediterranean deltas and coasts over the last 6000years: From progradation to destruction phase? Earth‐Science Reviews, 139, 336–361. 10.1016/j.earscirev.2014.10.003

[ece35049-bib-0009] Archibald, S. , Staver, A. C. , & Levin, S. A. (2012). Evolution of human‐driven fire regimes in Africa. Proceedings of the National Academy of Sciences of the United States of America, 109(3), 847–852. 10.1073/pnas.1118648109 22184249 PMC3271907

[ece35049-bib-0010] Armitage, D. K. , Plummer, R. , Berkes, F. , Arthur, R. I. , Charles, A. T. , Davidson‐Hunt, I. J. , … Wollenberg, E. K. (2009). Adaptive co‐management for social–ecological complexity. Frontiers in Ecology and the Environment, 7(2), 95–102.

[ece35049-bib-0011] Barlow, J. , Gardner, T. A. , Lees, A. C. , Parry, L. , & Peres, C. A. (2012). How pristine are tropical forests? An ecological perspective on the pre‐Columbian human footprint in Amazonia and implications for contemporary conservation. Biological Conservation, 151, 45–49. 10.1016/j.biocon.2011.10.013

[ece35049-bib-0012] Barrett, C. B. , Lee, D. R. , & McPeak, J. G. (2005). Institutional arrangements for rural poverty reduction and resource conservation. World Development, 33(2), 193–197. 10.1016/j.worlddev.2004.07.008

[ece35049-bib-0013] Barrios‐Garcia, M. N. , & Ballari, S. A. (2012). Impact of wild boar (*Sus scrofa*) in its introduced and native range: A review. Biological Invasions, 14, 2283–2300. 10.1007/s10530-012-0229-6

[ece35049-bib-0014] Bassotti, G. , & Villanacci, V. (2013). The control of defecation in humans: An evolutionary advantage? Techniques in Coloproctology, 17(6), 623–624. 23740030 10.1007/s10151-013-1037-4

[ece35049-bib-0015] Bastille‐Rousseau, G. , Fortin, D. , Dussault, C. , Courtois, R. , & Ouellet, J. P. (2011). Foraging strategies by omnivores: Are black bears actively searching for ungulate neonates or are they simply opportunistic predators? Ecography, 34(4), 588–596.

[ece35049-bib-0016] Batterbury, S. H. (2018). Political ecology In Castree N., Hulme M., & Proctor J. D. (Eds.), Companion to environmental studies (pp. 439–442). Abingdon, UK: Routledge, GSE Research.

[ece35049-bib-0017] Bauch, S. C. , Sills, E. O. , & Pattanayak, S. K. (2014). Have we managed to integrate conservation and development? ICDP impacts in the Brazilian Amazon. World Development, 64, S135–S148. 10.1016/j.worlddev.2014.03.009

[ece35049-bib-0018] Belcher, B. , Ruíz‐Pérez, M. , & Achdiawan, R. (2005). Patterns and trends in the use and management of commercial NTFPs: Implications for livelihoods and conservation. World Development, 33(9), 1435–1452.

[ece35049-bib-0019] Benyshek, D. C. , & Watson, J. T. (2006). Exploring the thrifty genotype's food‐shortage assumptions: A cross‐cultural comparison of ethnographic accounts of food security among foraging and agricultural societies. American Journal of Physical Anthropology, 131, 120–126. 10.1002/ajpa.20334 16485298

[ece35049-bib-0020] Berkes, F. , & Jolly, D. (2002). Adapting to climate change: Social‐ecological resilience in a Canadian western Arctic community. Conservation Ecology, 5(2), 18 10.5751/ES-00342-050218

[ece35049-bib-0021] Bharucha, Z. , & Pretty, J. (2010). The roles and values of wild foods in agricultural systems. Philosophical Transactions of the Royal Society B: Biological Sciences, 365(1554), 2913–2926. 10.1098/rstb.2010.0123 PMC2935111 20713393

[ece35049-bib-0022] Bishop, R. R. , Church, M. J. , & Rowley‐Conwy, P. A. (2015). Firewood, food and human niche construction: The potential role of Mesolithic hunter‐gatherers in actively structuring Scotland's woodlands. Quaternary Science Reviews, 108, 51–75. 10.1016/j.quascirev.2014.11.004

[ece35049-bib-0023] Blasco, R. , & Peris, J. F. (2012). A uniquely broad spectrum diet during the Middle Pleistocene at Bolomor Cave (Valencia, Spain). Quaternary International, 252, 16–31.

[ece35049-bib-0024] Bleige Bird, R. (2015). Disturbance, complexity, scale: New approaches to the study of human‐environment interactions. Annual Review of Anthropology, 44, 241–257.

[ece35049-bib-0025] Bleige Bird, R. , Codding, B. F. , & Bird, D. W. (2009). What explains differences in men's and women's production? Determinants of gendered foraging inequalities among Martu. Human Nature, 20, 105–129. 25526954 10.1007/s12110-009-9061-9

[ece35049-bib-0026] Bliege Bird, R. , & Nimmo, D. (2018). Restore the lost ecological functions of people. Nature Ecology & Evolution, 2, 1050–1052. 29867099 10.1038/s41559-018-0576-5

[ece35049-bib-0027] Blouch, R. A. (1988). Ecology and conservation of the Javan Warty Pig *Sus verrucosus *Muller, 1840. Biological Conservation, 43, 295–307.

[ece35049-bib-0028] Boivin, N. L. , Zeder, M. A. , Fuller, D. Q. , Crowther, A. , Larson, G. , Erlandson, J. M. , … Petraglia, M. D. (2016). Ecological consequences of human niche construction: Examining long‐term anthropogenic shaping of global species distributions. Proceedings of the National Academy of Sciences of the United States of America, 113(23), 6388–6396. 10.1073/pnas.1525200113 27274046 PMC4988612

[ece35049-bib-0029] Bonacic, C. , Feber, R. , & Macdonald, D. (2006). Capture of the vicuña (*Vicugna vicugna*) for sustainable use: Animal welfare implications. Biological Conservation, 129, 543550 10.1016/j.biocon.2005.11.021

[ece35049-bib-0030] Bond, W. J. , & Keeley, J. E. (2005). Fire as a global ‘herbivore': The ecology and evolution of flammable ecosystems. Trends in Ecology & Evolution, 20(7), 387–394. 16701401 10.1016/j.tree.2005.04.025

[ece35049-bib-0031] Bonhommeau, S. , Dubroca, L. , Le Pape, O. , Barde, J. , Kaplan, D. M. , Chassot, E. , & Nieblas, A.‐E. (2013). Eating up the world's food web and the human trophic level. Proceedings of the National Academy of Sciences of the United States of America, 110(51), 20617–20620. 10.1073/pnas.1305827110 24297882 PMC3870703

[ece35049-bib-0032] Booke, H. E. (1981). The conundrum of the stock concept—are nature and nurture definable in fishery science? Canadian Journal of Fisheries and Aquatic Sciences, 38(12), 1479–1480.

[ece35049-bib-0033] Borrini, G. , Kothari, A. , De Oviedo, G. F. , & Oviedo, G. (2004). Indigenous and local communities and protected areas: Towards equity and enhanced conservation: Guidance on policy and practice for co‐managed protected areas and community conserved areas (No. 11). IUCN.

[ece35049-bib-0034] Boschian, G. , & Saccà, D. (2015). In the elephant, everything is good: Carcass use and re-use at Castel di Guido (Italy). Quaternary International, 361, 288–296.

[ece35049-bib-0035] Boyle, A. E. , & Anderson, M. R. (1996). Human rights approaches to environmental protection. New York, NY: Clarendon Press.

[ece35049-bib-0036] Brandt, S. A. (1988). Early Holocene mortuary practices and hunter-gatherer adaptations in southern Somalia. World Archaeology, 20(1), 40–56. 16470993 10.1080/00438243.1988.9980055

[ece35049-bib-0037] Brown, R. T. , Agee, J. K. , & Franklin, J. F. (2004). Forest restoration and fire: Principles in the context of place. Conservation Biology, 18(4), 903–912. 10.1111/j.1523-1739.2004.521_1.x

[ece35049-bib-0038] Bruun, H. H. , & Fritzbøger, B. (2002). The past impact of livestock husbandry on dispersal of plant seeds in the landscape of Denmark. AMBIO: A Journal of the Human Environment, 31(5), 425–431. 10.1579/0044-7447-31.5.425 12374051

[ece35049-bib-0039] Burnside, W. R. , Brown, J. H. , Burger, O. , Hamilton, M. J. , Moses, M. , & Bettencourt, L. (2012). Human macroecology: Linking pattern and process in big‐picture human ecology. Biological Reviews, 87(1), 194–208. 10.1111/j.1469-185X.2011.00192.x 21781233

[ece35049-bib-0040] Byers, D. A. , & Broughton, J. M. (2004). Holocene environmental change, artiodactyl abundances, and human hunting strategies in the Great Basin. American Antiquity, 69(2), 235–255.

[ece35049-bib-0041] Cantrell, B. , Martin, L. J. , & Ellis, E. C. (2017). Designing autonomy: Opportunities for new wildness in the Anthropocene. Trends in Ecology & Evolution, 32(3), 156–166. 10.1016/j.tree.2016.12.004 28108135

[ece35049-bib-0042] Castilla, J. C. (1999). Coastal marine communities: Trends and perspectives from human‐exclusion experiments. Trends in Ecology & Evolution, 14(7), 280–283. 10370266 10.1016/s0169-5347(99)01602-x

[ece35049-bib-0043] Chalcraft, D. R. , & Resetarits, W. J. Jr (2003). Mapping functional similarity of predators on the basis of trait similarities. American Naturalist, 162(4), 390–402. 10.1086/378210 14582003

[ece35049-bib-0044] Chase‐Dunn, C. , & Lerro, B. (2013). Social change: Globalization from the Stone Age to the present. Boulder, CO: Paradigm.

[ece35049-bib-0045] Colautti, R. I. , & MacIsaac, H. J. (2004). A neutral terminology to define ‘invasive' species. Diversity and Distributions, 10(2), 135–141.

[ece35049-bib-0046] Collard, M. , & Wood, B. (2007). Hominin homoiology: An assessment of the impact of phenotypic plasticity on phylogenetic analyses of humans and their fossil relatives. Journal of Human Evolution, 52(5), 573–584. 10.1016/j.jhevol.2006.11.018 17412395

[ece35049-bib-0047] Comberti, C. , Thornton, T. F. , Echeverria, V. W. , & Patternson, T. (2015). Ecosystem services or services to ecosystems? Valuing cultivation and reciprocal relationships between humans and ecosystems. Global Environmental Change, 34, 247–262. 10.1016/j.gloenvcha.2015.07.007

[ece35049-bib-0048] Corlett, R. T. (2013). The shifted baseline: Prehistoric defaunation in the tropics and its consequences for biodiversity conservation. Biological Conservation, 163, 13–21. 10.1016/j.biocon.2012.11.012

[ece35049-bib-0049] Crutzen, P. J. (2002). Geology of mankind. Nature, 415(6867), 23–23. 10.1038/415023a 11780095

[ece35049-bib-0050] Cundill, G. , Cumming, G. S. , Biggs, D. , & Fabricius, C. (2011). Soft systems thinking and social learning for adaptive management. Conservation Biology, 26(1), 13–20. 22010884 10.1111/j.1523-1739.2011.01755.x

[ece35049-bib-0051] Dale, D. , & Ashley, C. Z. (2010). Holocene hunter-fisher-gatherer communities: New perspectives on Kansyore using communities of Western Kenya. Azania: Archaeological Research in Africa, 45(1), 24–48.

[ece35049-bib-0052] Darimont, C. T. , Fox, C. H. , Bryan, H. M. , & Reimchen, T. E. (2015). The unique ecology of human predators. Science, 349(6250), 858–860. 10.1126/science.aac4249 26293961

[ece35049-bib-0053] Diamond, J. (2002). Evolution, consequences and future of plant and animal domestication. Nature, 418(6898), 700–707. 12167878 10.1038/nature01019

[ece35049-bib-0054] Díaz, S. , Lavorel, S. , de Bello, F. , Quétier, F. , Grigulis, K. , & Robson, T. M. (2007). Incorporating plant functional diversity effects in ecosystem service assessments. Proceedings of the National Academy of Sciences of the United States of America, 104(52), 20684–20689. 10.1073/pnas.0704716104 18093933 PMC2410063

[ece35049-bib-0055] Dirzo, R. , Young, H. S. , Galetti, M. , Ceballos, G. , Isaac, N. J. , & Collen, B. (2014). Defaunation in the Anthropocene. Science, 345, 401–406. 25061202 10.1126/science.1251817

[ece35049-bib-0056] Donlan, J. C. , Berger, J. , Bock, C. E. , Bock, J. H. , Burney, D. A. , Estes, J. A. , … Soulé, M. E. (2006). Pleistocene rewilding: An optimistic agenda for twenty‐first century conservation. American Naturalist, 168(5), 660–681. 10.1086/508027 17080364

[ece35049-bib-0057] Elanchezhian, R. , Kumar, R. S. , Beena, S. J. , & Suryanarayana, M. A. (2007). Ethnobotany of Shompens–a primitive tribe of great Nicobar Island. Indian Journal of Traditional Knowledge, 6(2), 342–345.

[ece35049-bib-0058] Ellis, E. C. (2015). Ecology in an anthropogenic biosphere. Ecological Monographs, 85, 287–331. 10.1890/14-2274.1

[ece35049-bib-0059] Ellis, E. C. , Kaplan, J. O. , Fuller, D. Q. , Vavrus, S. , Goldewijk, K. K. , & Verburg, P. H. (2013). Used planet: A global history. Proceedings of the National Academy of Sciences of the United States of America, 110(20), 7978–7985. 10.1073/pnas.1217241110 23630271 PMC3657770

[ece35049-bib-0060] Ejrnæs, R. (2015). Step carefully, there is an elephant in the room: Human trampling as threat or treat in conservation. Applied Vegetation Science, 18(3), 357–358.

[ece35049-bib-0061] Estioko‐Griffin, A. (1986). Daughters of the forest. Agta women of the Philippines hunt large game animals and still raise their children. Natural History, 95(5), 36–43.

[ece35049-bib-0062] Faurby, S. , & Svenning, J.‐C. (2015). Historic and prehistoric human‐driven extinctions have reshaped global mammal diversity patterns. Diversity and Distributions, 21, 1155–1166. 10.1111/ddi.12369

[ece35049-bib-0063] Fergusen, S. H. , & McLoughlin, P. D. (2000). Effect of energy availability, seasonality, and geographic range on brown bear life history. Ecography, 23, 193–200. 10.1111/j.1600-0587.2000.tb00275.x

[ece35049-bib-0064] Ferraro, J. V. , Plummer, T. W. , Pobiner, B. L. , Oliver, J. S. , Bishop, L. C. , Braun, D. R. , Potts, R. (2013). Earliest archaeological evidence of persistent hominin carnivory. PLoS ONE, 8(4), e62174. 23637995 10.1371/journal.pone.0062174 PMC3636145

[ece35049-bib-0065] Feurdean, A. , Spessa, A. , Magyari, E. K. , Willis, K. J. , Veres, D. , & Hickler, T. (2012). Trends in biomass burning in the Carpathian region over the last 15,000 years. Quaternary Science Reviews, 45, 111–125. 10.1016/j.quascirev.2012.04.001

[ece35049-bib-0066] Foley, S. F. , Gronenborn, D. , Andreae, M. O. , Kadereit, J. W. , Esper, J. , Scholz, D. , … Crutzen, P. J. (2013). The Palaeoanthropocene‐The beginnings of anthropogenic environmental change. Anthropocene, 3, 83–88. 10.1016/j.ancene.2013.11.002

[ece35049-bib-0067] Folk, C. , Hahn, T. , Olsson, P. , & Norberg, J. (2005). Adaptive governance of social‐ecological systems. Annual Review of Environment and Resources, 30, 441–473. 10.1146/annurev.energy.30.050504.144511

[ece35049-bib-0068] Ford, J. K. B. , Ellis, G. M. , Barrett‐Lennard, L. G. , Morton, A. B. , Palm, R. S. , & Balcomb, K. C. III (1997). Dietary specialization in two sympatric populations of killer whales (*Orcinus orca*) in coastal British Columbia and adjacent waters. Canadian Journal of Zoology, 76, 1456–1471.

[ece35049-bib-0069] Foster, J. B. (1999). Marx's theory of metabolic rift: Classical foundations for environmental sociology. American Journal of Sociology, 105(2), 366–405. 10.1086/210315

[ece35049-bib-0070] Galetti, M. , Pires, A. S. , Brancalion, P. H. S. , & Fernandez, F. A. S. (2017). Reversing defaunation by trophic rewilding in empty forests. Biotropica, 49(1), 5–8. 10.1111/btp.12407

[ece35049-bib-0071] Gell, A. (1996). Vogel's Net: Traps as artworks and artworks as traps. Journal of Material Culture, 1, 15–38. 10.1177/135918359600100102

[ece35049-bib-0072] Gellner, G. , & McCann, K. (2011). Reconciling the omnivory-stability debate. The American Naturalist, 179(1), 22–37. 10.1086/663191 22173458

[ece35049-bib-0073] Gende, S. M. , Miller, A. E. , & Hood, E. (2007). The effects of salmon carcasses on soil nitrogen pools in a riparian forest of southeastern Alaska. Canadian Journal of Forest Research, 37, 1194–1202. 10.1139/X06-318

[ece35049-bib-0074] Gepts P., Famula T. R., Bettinger R. L., Brush S. B., Damania A. B., McGuire P. E., & Qualset C. O. (Eds.) (2012). Biodiversity in agriculture: Domestication, evolution, and sustainability. Cambridge, UK: Cambridge University Press.

[ece35049-bib-0075] Ghiglieri, M. P. , Butynski, T. M. , & Struhsaker, T. T. (1982). Bush pig (*Potamochoerus porcus*) polychromism and ecology in Kibale Forest, Uganda. African Journal of Ecology, 20, 233–236.

[ece35049-bib-0076] Gibson‐Graham, J. K. (2006). A postcapitalist politics. Minneapolis, MN: University of Minnesota Press.

[ece35049-bib-0077] Gilbert, S. F. (2001). Ecological developmental biology: Developmental biology meets the real world. Developmental Biology, 233(1), 1–12. 11319853 10.1006/dbio.2001.0210

[ece35049-bib-0078] Gilbert, S. F. , & Epel, D. (2009). Ecological developmental biology: Integrating epigenetics, medicine, and evolution. Sunderland, MA: Sinauer Associates.

[ece35049-bib-0079] Gill, J. L. , Williams, J. W. , Jackson, S. T. , Lininger, K. B. , & Robinson, G. S. (2009). Pleistocene megafaunal collapse, novel plant communities, and enhanced fire regimes in North America. Science, 326(5956), 1100–1103. 10.1126/science.1179504 19965426

[ece35049-bib-0080] Grayson, D. K. (2001). The archaeological record of human impacts on animal populations. Journal of World Prehistory, 15(1), 1–68.

[ece35049-bib-0081] Gremillion, K. J. , Barton, L. , & Piperno, D. R. (2014). Particularism and the retreat from theory in the archaeology of agricultural origins. Proceedings of the National Academy of Sciences of the United States of America, 111(17), 6171–6177. 10.1073/pnas.1308938110 24753601 PMC4035987

[ece35049-bib-0082] Griffiths, C. J. , Zuel, N. , Jones, C. G. , Ahamud, Z. , & Harris, S. (2013). Assessing the potential to restore historic grazing ecosystems with tortoise ecological replacements. Conservation Biology, 27(4), 690–700. 10.1111/cobi.12087 23773124

[ece35049-bib-0083] Grigg, D. B. (1974). The agricultural systems of the world: An evolutionary approach (Vol. 5). Cambridge, UK: Cambridge University Press.

[ece35049-bib-0084] Guimarães, P. R. Jr , Galetti, M. , & Jordano, P. (2008). Seed dispersal anachronisms: Rethinking the fruits extinct megafauna ate. PLoS ONE, 3(3), e1745 10.1371/journal.pone.0001745 18320062 PMC2258420

[ece35049-bib-0085] Hansen, D. M. , & Galetti, M. (2009). The forgotten megafauna. Science, 324, 42–43. 10.1126/science.1172393 19342573

[ece35049-bib-0086] Hardy, B. L. , & Moncel, M. H. (2011). Neanderthal use of fish, mammals, birds, starchy plants and wood 125–250,000 years ago. PLoS ONE, 6(8), e23768. 21887315 10.1371/journal.pone.0023768 PMC3161061

[ece35049-bib-0087] Harris, D. R. (2012). Evolution of agroecosystems: biodiversity, origins and differential development In Gepts P., et al. (Eds.), Biodiversity in agriculture: Domestication, evolution and sustainability (pp. 21–56). Cambridge, UK: Cambridge University Press.

[ece35049-bib-0088] Hawkes, K. , & Coxworth, J. E. (2013). Grandmothers and the evolution of human longevity: A review of findings and future directions. Evolutionary Anthropology, 22, 294–302. 24347503 10.1002/evan.21382

[ece35049-bib-0089] Hebblewhite, M. , & Merrill, E. H. (2007). Multiscale wolf predation risk for elk: Does migration reduce risk? Oecologia, 152, 377–387. 17287955 10.1007/s00442-007-0661-y

[ece35049-bib-0090] Hebblewhite, M. , & Merrill, E. H. (2009). Trade‐offs between predation risk and forage differ between migrant strategies in a migratory ungulate. Ecology, 90, 3445–3454. 10.1890/08-2090.1 20120812

[ece35049-bib-0091] Heinken, T. , Schmidt, M. , von Oheimb, G. , Kriebitzsch, W.‐U. , & Ellenberg, H. (2006). Soil seed banks near rubbing trees indicate dispersal of plant species into forests by wild boar. Basic and Applied Ecology, 7, 31–44. 10.1016/j.baae.2005.04.006

[ece35049-bib-0092] Henry, A. G. , Brooks, A. S. , & Piperno, D. R. (2014). Plant foods and the dietary ecology of Neanderthals and early modern humans. Journal of Human Evolution, 69, 44–54. 24612646 10.1016/j.jhevol.2013.12.014

[ece35049-bib-0093] Holdaway, R. N. , Allentoft, M. E. , Jacomb, C. , Oskam, C. L. , Beavan, N. R. , & Bunce, M. (2014). An extremely low‐density human population exterminated New Zealand moa. Nature Communications, 5, 5436. 10.1038/ncomms6436 25378020

[ece35049-bib-0094] Holtgrieve, G. W. , Schindler, D. E. , & Jewett, P. K. (2009). Large predators and biogeochemical hotspots: Brown bear (*Ursus arctos*) predation on salmon alters nitrogen cycling in riparian soils. Ecological Research, 24, 1125–1135. 10.1007/s11284-009-0591-8

[ece35049-bib-0095] Hortal, J. , de Bello, F. , Diniz‐Filho, J. A. F. , Lewinsohn, T. M. , Lobo, J. M. , & Ladle, R. J. (2015). Seven shortfalls that beset large‐scale knowledge of biodiversity. Annual Review of Ecology, Evolution, and Systematics, 46, 523–549. 10.1146/annurev-ecolsys-112414-054400

[ece35049-bib-0096] Infield, M. , Entwistle, A. , Anthem, H. , Mugisha, A. , & Phillips, K. (2018). Reflections on cultural values approaches to conservation: Lessons from 20 years of implementation. Oryx, 52(2), 220–230. 10.1017/S0030605317000928

[ece35049-bib-0097] Ingold, T. (1980). Hunters, pastoralists and ranchers: Reindeer economies and their transformations. New York, NY: Cambridge University Press.

[ece35049-bib-0098] Ito, T. Y. , Lhagvasuren, B. , Tsunekawa, A. , Shinoda, M. , Takatsuki, S. , Buuveibaatar, B. , & Chimeddorj, B. (2013). Fragmentation of the habitat of wild ungulates by anthropogenic barriers in Mongolia. PLoS ONE, 8(2), e56995 10.1371/journal.pone.0056995 23437291 PMC3577783

[ece35049-bib-0099] Jablonka, E. , & Lamb, M. J. (2007). Précis of evolution in four dimensions. The Behavioral and Brain Sciences, 30(4), 353–365. 10.1017/S0140525X07002221 18081952

[ece35049-bib-0100] Jachowski, D. S. , Kesler, D. C. , Steen, D. A. , & Walters, J. R. (2015). Redefining baselines in endangered species recovery. Journal of Wildlife Management, 79(1), 3–9.

[ece35049-bib-0101] Janzen, D. H. (2001). Latent extinction—the living dead In Levin S. A. (Ed.), Encyclopedia of biodiversity (Vol. 3, pp. 689–699). Cambridge, MA: Academic Press.

[ece35049-bib-0102] Jesmer, B. R. , Merkle, J. A. , Goheen, J. R. , Aikens, E. O. , Beck, J. L. , Courtemanch, A. B. , … Kauffman, M. J. (2018). Is ungulate migration culturally transmitted? Evidence of social learning from translocated animals. Science, 361(6406), 1023–1025. 10.1126/science.aat0985 30190405

[ece35049-bib-0103] Johnson, C. N. (2009). Ecological consequences of Late Quaternary extinctions of megafauna. Proceedings of the Royal Society B: Biological Sciences, 276(1167), 2509–2520. 10.1098/rspb.2008.1921 PMC2684593 19324773

[ece35049-bib-0104] Johnson, C. N. , Prior, L. D. , Archibald, S. , Poulos, H. M. , Barton, A. M. , Williamson, G. J. , & Bowman, D. M. J. S. (2018). Can trophic rewilding reduce the impact of fire in a more flammable world? Philosophical Transactions of the Royal Society B: Biological Sciences, 373, 20170443. 10.1098/rstb.2017.0443 PMC6231065 30348870

[ece35049-bib-0105] Jones, J. H. (2015). Resource transfers and human life history evolution. Annual Review of Anthropology, 44, 513–531. 10.1146/annurev-anthro-102214-013938

[ece35049-bib-0106] Jones, K. E. , Bielby, J. , Cardillo, M. , Fritz, S. A. , O'Dell, J. , Orme, C. D. L. , … Purvis, A. (2009). PanTHERIA: A species‐level database of life history, ecology, and geography of extant and recently extinct mammals. Ecology, 90, 2648.

[ece35049-bib-0107] Kay, C. E. (1994). Aboriginal overkill. Human Nature, 5, 359–398. 10.1007/BF02734166 24214685

[ece35049-bib-0108] Keller, A. (2007). Drosophila melanogaster's history as a human commensal. Current Biology, 17(3), R77–R81. 10.1016/j.cub.2006.12.031 17276902

[ece35049-bib-0109] Kelly, R. L. (1995). The foraging spectrum: Diversity in hunter‐gatherer lifeways. Washington, DC: Smithsonian Institution Press.

[ece35049-bib-0110] Kissling, W. D. , Dalby, L. , Fløjgaard, C. , Lenoir, J. , Sandel, B. , Sandom, C. , … Svenning, J.‐C. (2014). Establishing macroecological trait datasets: Digitalization, extrapolation, and validation of diet preferences in terrestrial mammals worldwide. Ecology and Evolution, 4, 2913–2930. 10.1002/ece3.1136 25165528 PMC4130448

[ece35049-bib-0111] Kistler, L. , Newsom, L. A. , Ryan, T. M. , Clarke, A. C. , Smith, B. D. , & Perry, G. H. (2015). Gourds and squashes (*Cucurbita* spp.) adapted to megafaunal extinction and ecological anachronism through domestication. Proceedings of the National Academy of Sciences of the United States of America, 112(49), 15107–15112. 26630007 10.1073/pnas.1516109112 PMC4679018

[ece35049-bib-0112] Klein, R. (2005). Hominin dispersal in the Old World In Scarre C. (Ed.), The human past: World prehistory and the development of human societies (pp. 84–123). London, UK: Thames & Hudson.

[ece35049-bib-0113] Koch, P. L. , & Barnosky, A. D. (2006). Late Quaternary extinctions: State of the debate. Annual Review of Ecology, Evolution, and Systematics, 37, 215–250. 10.1146/annurev.ecolsys.34.011802.132415

[ece35049-bib-0114] Krader, L. (1955). Ecology of Central Asian Pastoralism. Southwestern Journal of Anthropology, 11(4), 301–326. 10.1086/soutjanth.11.4.3628907

[ece35049-bib-0115] Kraft, T. S. , Venkataraman, V. V. , & Dominy, N. J. (2014). A natural history of human tree climbing. Journal of Human Evolution, 71, 105–118. 24630525 10.1016/j.jhevol.2014.02.002

[ece35049-bib-0116] Kratina, P. , LeCraw, R. M. , Ingram, T. , & Anholt, B. R. (2012). Stability and persistence of food webs with omnivory: Is there a general pattern? Ecosphere, 3(6), art50.

[ece35049-bib-0117] Krausmann, F. , Erb, K.‐h. , Gingrich, S. , Haberl, H. , Bondeau, A. , Gaube, V. , … Searchinger, T. d. (2013). Global human appropriation of net primary production doubled in the 20th century. Proceedings of the National Academy of Sciences of the United States of America, 110(25), 10324–10329. 10.1073/pnas.1211349110 23733940 PMC3690849

[ece35049-bib-0118] Kuhn, S. , Stiner, M. , Bar Oz, G. , Weinstein Evron, M. , Bocquet Appel, J. , Hovers, E. , … Stiner, M. C. (2006). What's a mother to do? The division of labor among Neandertals and modern humans in Eurasia. Current Anthropology, 47(6), 953–981. 10.1086/507197

[ece35049-bib-0119] Kuneš, P. , Pokorný, P. , & Šída, P. (2008). Detection of the impact of early Holocene hunter‐gatherers on vegetation in the Czech Republic, using multivariate analysis of pollen data. Vegetation History and Archaeobotany, 17, 269–287. 10.1007/s00334-007-0119-5

[ece35049-bib-0120] Kuussaari, M. , Bommarco, R. , Heikkinen, R. K. , Helm, A. , Krauss, J. , Lindborg, R. , … Steffan‐Dewenter, I. (2009). Extinction debt: A challenge for biodiversity conservation. Trends in Ecology & Evolution, 24(10), 564–571. 10.1016/j.tree.2009.04.011 19665254

[ece35049-bib-0121] Larson, G. , Karlsson, E. k. , Perri, A. , Webster, M. t. , Ho, S. y. w. , Peters, J. , … Lindblad‐Toh, K. (2012). Rethinking dog domestication by integrating genetics, archeology, and biogeography. Proceedings of the National Academy of Sciences of the United States of America, 109(23), 8878–8883. 10.1073/pnas.1203005109 22615366 PMC3384140

[ece35049-bib-0122] Laurie, A. , & Seidensticker, J. (1977). Behavioral ecology of the Sloth bear (*Melursus ursinus*). Journal of Zoology, 182, 187–204.

[ece35049-bib-0123] LeCount, A. L. (1980). Some aspects of black bear ecology in the Arizona chaparral. Bears: Their Biology and Management, 4, 175–179. 10.2307/3872864

[ece35049-bib-0124] Lee, C. E. (2002). Evolutionary genetics of invasive species. Trends in Ecology & Evolution, 17(8), 386–391.

[ece35049-bib-0125] Leus, K. , & MacDonald, A. (1997). From babirusa (*Babyrousa babyrussa*) to domestic pig: The nutrition of swine. Proceedings of the Nutrition Society, 56, 1001–1012. 10.1079/PNS19970105 9483666

[ece35049-bib-0126] Lieberman, D. E. , & Bramble, D. M. (2007). The evolution of marathon running. Sports Medicine, 37(4–5), 288–290. 17465590 10.2165/00007256-200737040-00004

[ece35049-bib-0127] Loo, S. L. , Hawkes, K. , & Kim, P. S. (2017). Evolution of male strategies with sex‐ratio–dependent pay‐offs: Connecting pair bonds with grandmothering. Philosophical Transactions of the Royal Society B: Biological Sciences, 372, 20170041. 10.1098/rstb.2017.0041 PMC5540867 28760768

[ece35049-bib-0128] Lorimer, J. , Sandom, C. , Jepson, P. , Doughty, C. , Barua, M. , & Kirby, K. J. (2015). Rewilding: Science, practice and politics. Annual Review of Environment and Resources, 40, 39–62. 10.1146/annurev-environ-102014-021406

[ece35049-bib-0129] Lundberg, J. , & Moberg, F. (2003). Mobile link organisms and ecosystem functioning: Implications for ecosystem resilience and management. Ecosystems, 6, 87–98. 10.1007/s10021-002-0150-4

[ece35049-bib-0130] Lundholm, J. T. , & Richardson, P. J. (2010). Habitat analogues for reconciliation ecology in urban and industrial environments. Journal of Applied Ecology, 47, 966–976.

[ece35049-bib-0131] Macho, G. A. (2014). Baboon feeding ecology informs the dietary niche of Paranthropus boisei. PLoS ONE, 9(1), e84942. 24416315 10.1371/journal.pone.0084942 PMC3885648

[ece35049-bib-0132] Manner, H. I. (1981). Ecological succession in new and old swiddens of montane Papua New Guinea. Human Ecology, 9(3), 359–377.

[ece35049-bib-0133] Marlowe, F. W. (2005). Hunter‐gatherers and human evolution. Evolutionary Anthropology: Issues, News, and Reviews, 14(2), 54–67. 10.1002/evan.20046

[ece35049-bib-0134] Martin, P. S. (1984). Prehistoric overkill: The global model In Martin P. S., & Klein R. G. (Eds.), Quaternary extinctions: A prehistoric revolution. Tuscon, AZ: University of Arizona Press.

[ece35049-bib-0135] Martin, P. S. (1970). Pleistocene niches for alien animals. BioScience, 20, 218–221. 10.2307/1295128

[ece35049-bib-0136] Martin, P. S. , & Szuter, C. R. (1999). War zones and game sinks in Lewis and Clark's West. Conservation Biology, 13, 36–45. 10.1046/j.1523-1739.1999.97417.x

[ece35049-bib-0137] Mason, S. , Newsome, D. , Moore, S. , & Admiraal, R. (2015). Recreational trampling negatively impacts vegetation structure of an Australian biodiversity hotspot. Biodiversity and Conservation, 24, 2685–2707. 10.1007/s10531-015-0957-x

[ece35049-bib-0138] McConkey, K. , & Galetti, M. (1999). Seed dispersal by the Sun Bear *Helarctos malayanus* in Central Borneo. Journal of Tropical Ecology, 15(2), 237–241. 10.1017/S0266467499000784

[ece35049-bib-0139] McMichael, C. H. , Piperno, D. R. , Bush, M. B. , Silman, M. R. , Zimmerman, A. R. , Raczka, M. F. , & Lobato, L. C. (2012). Sparse pre‐Columbian human habitation in Western Amazonia. Science, 336(1429), 1219982 10.1126/science.1219982 22700926

[ece35049-bib-0140] Michon, G. , De Foresta, H. , Levang, P. , & Verdeaux, F. (2007). Domestic forests: A new paradigm for integrating local communities' forestry into tropical forest science. Ecology and Society, 12(2), 1.

[ece35049-bib-0141] Milder, J. C. , Hart, A. K. , Dobie, P. , Minai, J. , & Zaleski, C. (2014). Integrated landscape initiatives for African agriculture, development and conservation: A region‐wide assessment. World Development, 54, 68–80. 10.1016/j.worlddev.2013.07.006

[ece35049-bib-0142] Miller, S. D. (1990). Denning ecology of brown bears in Southcentral Alaska and comparisons with a sympatric black bear population. Bears: Their Biology and Management, 8, 279–287. 10.2307/3872930

[ece35049-bib-0143] Montes, M. C. , Carmanchahi, P. D. , Rey, A. , & Funes, M. C. (2006). Live shearing free‐ranging guanacos (*Lama guanicoe*) in Patagonia for sustainable use. Journal of Arid Environments, 64, 616–625. 10.1016/j.jaridenv.2005.05.008

[ece35049-bib-0144] Morelle, K. , Podgórski, T. , Prévot, C. , Keuling, O. , Lehaire, F. , & Lejeune, P. (2014). Towards understanding wild boar Sus scrofa movement: A synthetic movement ecology approach. Mammal Review, 45, 15–29.

[ece35049-bib-0145] Muler, A. E. , Rother, D. C. , Brancalion, P. S. , Naves, R. P. , Rodrigues, R. R. , & Pizo, M. A. (2014). Can overharvesting of a non‐timber‐forest‐product change the regeneration dynamics of a tropical rainforest? The case study of Euterpe edulis. Forest Ecology and Management, 324, 117–125. 10.1016/j.foreco.2013.09.001

[ece35049-bib-0146] Munro, R. H. M. , Nielsen, S. E. , Price, M. H. , Stenhouse, G. B. , & Boyce, M. S. (2006). Seasonal and diel patterns of grizzly bear diet and activity in West‐Central Alberta. Journal of Mammalogy, 87(6), 1112–1121.

[ece35049-bib-0147] Naundrup, P. J. , & Svenning, J. C. (2015). A geographic assessment of the global scope for rewilding with wild‐living horses (*Equus ferus*). PLoS ONE, 10(7), e0132359. 26177104 10.1371/journal.pone.0132359 PMC4503665

[ece35049-bib-0148] Navarro, L. M. , & Pereira, H. M. (2015). Rewilding abandoned landscapes in Europe In Rewilding European landscapes (pp. 3–23). Cham, Switzerland: Springer.

[ece35049-bib-0149] Niven, L. , Steele, T. E. , Rendu, W. , Mallye, J.‐B. , McPherron, S. P. , Soressi, M. , … Hublin, J.‐J. (2012). Neandertal mobility and large‐game hunting: The exploitation of reindeer during the Quina Mousterian at Chez‐Pinaud Jonzac (Charente‐Maritime, France). Journal of Human Evolution, 63, 624–635. 10.1016/j.jhevol.2012.07.002 22951376

[ece35049-bib-0150] Nogues‐Bravo, D. , Simberloff, D. , Rahbek, C. , & Sanders, N. J. (2016). Rewilding is the new Pandora's box in conservation. Current Biology, 26, R83–R101. 10.1016/j.cub.2015.12.044 26859272

[ece35049-bib-0151] Norkko, A. , Villnas, A. , Norkko, J. , Valanko, S. , & Pilditch, C. (2013). Size matters: Implications of the loss of large individuals for ecosystem function. Scientific Reports, 3, 2646. 24025973 10.1038/srep02646 PMC6505624

[ece35049-bib-0152] Noyce, K. V. , Kannowski, P. B. , & Riggs, M. R. (1997). Black bears as ant‐eaters: Seasonal associations between bear myrmecophagy and ant ecology in north‐central Minnesota. Canadian Journal of Zoology, 75, 1671–1686. 10.1139/z97-794

[ece35049-bib-0153] Oswalt W. (Ed.). (1972). Modern Alaskan Native material culture. Fairbanks, AK: University of Alaska Museum.

[ece35049-bib-0154] Papworth, S. K. , Rist, J. , Coad, L. , & Milner‐Gulland, E. J. (2009). Evidence for shifting baseline syndrome in conservation. Conservation Letters, 2(2), 93–100. 10.1111/j.1755-263X.2009.00049.x

[ece35049-bib-0155] Pearson, K. L. (1997). Nutrition and the early‐medieval diet. Speculum, 72(1), 1–32. 10.2307/2865862

[ece35049-bib-0156] Pérez‐Méndez, N. , Jordano, P. , & Valido, A. (2015). Downsized mutualisms: Consequences of seed dispersers' body‐size reduction for early plant recruitment. Perspectives in Plant Ecology, Evolution and Systematics, 17, 151–159.

[ece35049-bib-0157] Pickering, T. R. (2006). Subsistence behaviour of South African Pleistocene hominids. South African Journal of Science, 102(5), 205–210.

[ece35049-bib-0158] Pickering, T. R. , & Bunn, H. T. (2007). The endurance running hypothesis and hunting and scavenging in Savanna‐woodlands. Journal of Human Evolution, 53(4), 434–438. 17720224 10.1016/j.jhevol.2007.01.012

[ece35049-bib-0159] Piersma, T. , & Van Gils, J. A. (2011). The flexible phenotype: A body‐centred integration of ecology, physiology, and behaviour. Oxford, UK: Oxford University Press.

[ece35049-bib-0160] Pires, M. M. , Galetti, M. , Donatti, C. I. , Pizo, M. A. , Dirzo, R. , & Guimarães, P. R. (2014). Reconstructing past ecological networks: The reconfiguration of seed-dispersal interactions after megafaunal extinction. Oecologia, 175(4), 1247–1256. 24865393 10.1007/s00442-014-2971-1

[ece35049-bib-0161] Poschlod, P. , & Bonn, S. (1998). Changing dispersal processes in the central European landscape since the last ice age: An explanation for the actual decrease of plant species richness in different habitats? Acta Botanica Neerlandica, 47, 27–44.

[ece35049-bib-0162] Putman, R. , & Flueck, W. T. (2011). Intraspecific variation in biology and ecology of deer: Magnitude and causation. Animal Production Science, 51, 277–291.

[ece35049-bib-0163] Rantala, M. J. (2007). Evolution of nakedness in Homo sapiens. Journal of Zoology, 273, 1–7.

[ece35049-bib-0164] Rapport, D. , & Maffi, L. (2010). The dual erosion of biological and cultural diversity: Implications for the health of ecocultural systems In Pilgrim S., & Pretty J. N. (Eds.), Nature and culture: Rebuilding lost connections (pp. 103–123). London, UK: Earthscan.

[ece35049-bib-0165] Reid, R. S. , Nkedianye, D. , Said, M. Y. , Kaelo, D. , Neselle, M. , Makui, O. , … Clark, W. C. (2016). Evolution of models to support community and policy action with science: Balancing pastoral livelihoods and wildlife conservation in savannas of East Africa. Proceedings of the National Academy of Sciences of the United States of America, 113(17), 4579–4584. 10.1073/pnas.0900313106 19887640 PMC4855612

[ece35049-bib-0166] Reinhard, K. J. , Hevly, R. H. , & Anderson, G. A. (1987). Helminth remains from prehistoric Indian coprolites on the Colorado Plateau. The Journal of Parasitology, 73(3), 630–639. 3298603

[ece35049-bib-0167] Richards, M. (2002). A brief review of the archaeological evidence for Palaeolithic and Neolithic subsistence. European Journal of Clinical Nutrition, 56, 1262–1278. 10.1038/sj.ejcn.1601646 12494313

[ece35049-bib-0168] Richards, M. P. , & Trinkaus, E. (2009). Isotopic evidence for the diets of European Neanderthals and early modern humans. Proceedings of the National Academy of Sciences of the United States of America, 106, 16034–16039. 10.1073/pnas.0903821106 19706482 PMC2752538

[ece35049-bib-0169] Ripple, W. J. , Estes, J. A. , Beschta, R. l. , Wilmers, C. C. , Ritchie, E. G. , Hebblewhite, M. , … Wirsing, A. J. (2014). Status and ecological effects of the world's largest carnivores. Science, 343(6167), 1241484 10.1126/science.1241484 24408439

[ece35049-bib-0170] Ripple, W. J. , Newsome, T. M. , Wolf, C. , Dirzo, R. , Everatt, K. T. , Galetti, M. , … Van Valkenburgh, B. (2015). Collapse of the world's largest herbivores. Science Advances, 1(4), e1400103 10.1126/sciadv.1400103 26601172 PMC4640652

[ece35049-bib-0171] Roebroeks, W. , & Villa, P. (2011). On the earliest evidence for habitual use of fire in Europe. Proceedings of the National Academy of Sciences of the United States of America, 108(13), 5209–5214. 10.1073/pnas.1018116108 21402905 PMC3069174

[ece35049-bib-0172] Roelvink, G. , St. Martin, K. , & Gibson‐Graham, J. K. (Eds.) (2015). Making other worlds possible: Performing diverse economies. Minneapolis, MN: University of Minnesota Press.

[ece35049-bib-0173] Root‐Bernstein, M. (2013). Predicting the direction and magnitude of small mammal disturbance effects on plant diversity across scales. Frontiers of Biogeography, 5(2), 113–121. 10.21425/F55215278

[ece35049-bib-0174] Root‐Bernstein, M. , Galetti, M. , & Ladle, R. J. (2017). Rewilding South America: Ten key questions. Perspectives in Ecology and Conservation, 15(4), 271–281. 10.1016/j.pecon.2017.09.007

[ece35049-bib-0175] Root‐Bernstein, M. , Guerrero‐Gattica, M. , Piña, L. , Bonacic, C. , Svenning, J.‐C. , & Jaksic, F. M. (2016). Prospects for a model of rewilding‐inspired transhumance for the restoration of a semi‐arid silvopastoral system. Regional Environmental Change, 130, 54–61.

[ece35049-bib-0176] Root‐Bernstein, M. , & Svenning, J.‐C. (2016). Prospects for rewilding with camelids. Journal of Arid Environments, 130, 54–61.

[ece35049-bib-0177] Root‐Bernstein, M. , & Svenning, J.‐C. (2018). Human paths have positive impacts on plant richness and diversity: A meta‐analysis. Ecology and Evolution, 8, 11111–11121. 30519429 10.1002/ece3.4578 PMC6262937

[ece35049-bib-0178] Ross, E. M. , Moate, P. J. , Marett, L. C. , Cocks, B. G. , & Hayes, B. J. (2013). Metagenomic predictions: From microbiome to complex health and environmental phenotypes in humans and cattle. PLoS ONE, 8(9), e73056 10.1371/journal.pone.0073056 24023808 PMC3762846

[ece35049-bib-0179] Rowley‐Conwy, P. , & Layton, R. (2011). Foraging and farming as niche construction: Stable and unstable adaptations. Philosophical Transactions of the Royal Society B: Biological Sciences, 366(1566), 849–862. 10.1098/rstb.2010.0307 PMC3048996 21320899

[ece35049-bib-0180] Rubenstein, D. R. , Rubenstein, D. I. , Sherman, P. W. , & Gavin, T. A. (2006). Pleistocene Park: Does re-wilding North America represent sound conservation for the 21st century? Biological Conservation, 132(2), 232–238.

[ece35049-bib-0181] Rule, S. , Brook, B. W. , Haberle, S. G. , Turney, C. S. , Kershaw, A. P. , & Johnson, C. N. (2012). The aftermath of megafaunal extinction: Ecosystem transformation in Pleistocene Australia. Science, 335(6075), 1483–1486. 10.1126/science.1214261 22442481

[ece35049-bib-0182] Sanderson, E. W. , Redford, K. H. , Weber, B. , Aune, K. , Baldes, D. , Berger, J. , … Fearn, E. V. A. (2008). The ecological future of the North American Bison. Conservation Biology, 22(2), 252–266. 18402580 10.1111/j.1523-1739.2008.00899.x

[ece35049-bib-0183] Sandom, C. , Faurby, S. , Sandel, B. , & Svenning, J.‐C. (2014). Global late Quaternary megafauna extinctions linked to humans, not climate change. Proceedings of the Royal Society B: Biological Sciences, 281, 20133254 10.1098/rspb.2013.3254 PMC4071532 24898370

[ece35049-bib-0184] Sandom, C. J. , Hughes, J. , & Macdonald, D. W. (2012). Rewilding the scottish highlands: Do wild boar, sus scrofa, use a suitable foraging strategy to be effective ecosystem engineers? Restoration Ecology, 21(3), 336–343.

[ece35049-bib-0185] Saunders, G. , & Giles, J. (1995). Ecological comparison of two wild pig populations in semi‐arid and sub‐alpine Australia. Ibex, 3, 152–155.

[ece35049-bib-0186] Sayre, N. F. , McAllister, R. R. J. , Bestelmeyer, B. T. , Moritz, M. , & Turner, M. D. (2013). Earth Stewardship of rangelands: Coping with ecological, economic, and political marginality. Frontiers in Ecology and the Environment, 11(7), 348–354.

[ece35049-bib-0187] Schmidt, O. (2006). Wood and tree fungi: biology, damage, protection, and use. Berlin, Germany: Springer Science & Business Media.

[ece35049-bib-0188] Schmidt, M. (2013). Amazonian Dark Earths: Pathways to sustainable development in tropical rainforests? Boletim do Museu Paraense Emílio Goeldi Ciências Humanas, 8(11), 11–38.

[ece35049-bib-0189] Searcy, C. A. , Rollins, H. B. , & Shaffer, H. B. (2016). Ecological equivalency as a tool for endangered species management. Ecological Applications, 26(1), 94–103. 10.1890/14-1674 27039512

[ece35049-bib-0190] Seddon, P. J. , Griffiths, C. J. , Soorae, P. S. , & Armstrong, D. P. (2014). Reversing defaunation: Restoring species in a changing world. Science, 345(6195), 406–412. 25061203 10.1126/science.1251818

[ece35049-bib-0191] Servheen, C. (1983). Grizzly bear food habits, movements and habitat selection in the Mission Mountains, Montana. The Journal of Wildlife Management, 47(4), 1026–1035. 10.2307/3808161

[ece35049-bib-0192] Shaner, P.‐J.‐L. , & Macko, S. A. (2011). Trophic shifts of a generalist consumer in response to resource pulses. PLoS ONE, 6(3), e17970. 21437248 10.1371/journal.pone.0017970 PMC3060883

[ece35049-bib-0193] Shea, K. , Roxburgh, S. H. , & Rauscher, E. S. J. (2004). Moving from pattern to process: Coexistence mechanisms under intermediate disturbance regimes. Ecology Letters, 7, 491–508. 10.1111/j.1461-0248.2004.00600.x

[ece35049-bib-0194] Skinner, J. D. , Breytenbach, G. J. , & Maberly, C. T. A. (1976). Observations on the ecology and biology of the bushpig *Potamochoerus porcus* Linn. in the Northern Transvaal. South African Journal of Wildlife Research, 6(2), 123–128.

[ece35049-bib-0195] Stiner, M. C. (2002). Carnivory, coevolution, and the geographic spread of the genus Homo. Journal of Archaeological Research, 10(1), 1–63.

[ece35049-bib-0196] Stirling, I. , & Derocher, A. E. (1990). Factors affecting the evolution and behavioral ecology of the modern bears. Bears: Their Biology and Management, 8, 189–204. 10.2307/3872919

[ece35049-bib-0197] Stouffer, D. B. , & Bascompte, J. (2010). Understanding food-web persistence from local to global scales. Ecology Letters, 13(2), 154–161. 19968697 10.1111/j.1461-0248.2009.01407.x

[ece35049-bib-0198] Stripple, J. , & Bulkeley, H. (2015). Governmentality In Bäckstrand K., & Lövbrand E. (Eds.), Research handbook on climate governance (pp. 49–59). Cheltenham, UK: Edward Elgar Publishing.

[ece35049-bib-0199] Stuart, A. J. (1991). Mammalian extinctions in the Late Pleistocene of northern Eurasia and North America. Biological Reviews, 66(4), 453–562. 10.1111/j.1469-185X.1991.tb01149.x 1801948

[ece35049-bib-0200] Sullivan, A. P. , Bird, D. W. , & Perry, G. H. (2017). Human behaviour as a long‐term ecological driver of non‐human evolution. Nature Ecology & Evolution, 1, 65. 28812734 10.1038/s41559-016-0065

[ece35049-bib-0201] Sunderlin, W. D. , Angelsen, A. , Belcher, B. , Burgers, P. , Nasi, R. , Santoso, L. , & Wunder, S. (2005). Livelihoods, forests, and conservation in developing countries: An overview. World Development, 33(9), 1383–1402.

[ece35049-bib-0202] Svenning, J. C. , Pedersen, P. B. , Donlan, C. J. , Ejrnæs, R. , Faurby, S. , Galetti, M. , … Vera, F. W. (2015). Science for a wilder Anthropocene: Synthesis and future directions for trophic rewilding research. Proceedings of the National Academy of Sciences of the United States of America, 113, 898–906. 26504218 10.1073/pnas.1502556112 PMC4743824

[ece35049-bib-0203] Svizzero, S. , & Tisdell, C. A. (2015). The persistence of hunting and gathering economies. Social Evolution and History, 14(2), 3–25.

[ece35049-bib-0204] Swarts, N. D. , & Dixon, K. W. (2009). Terrestrial orchid conservation in the age of extinction. Annals of Botany, 104(3), 543–556. 19218582 10.1093/aob/mcp025 PMC2720663

[ece35049-bib-0205] Tardiff, S. E. , & Stanford, J. A. (1998). Grizzly bear digging: Effects on subalpine meadow plants in relation to mineral nitrogen availability. Ecology, 79, 2219–2228. 10.1890/0012-9658(1998)079[2219:GBDEOS]2.0.CO;2

[ece35049-bib-0206] Taylor, P. (2009). Re‐wildling the grazers: Obstacles to the “wild” in wildlife management. British Wildlife, 20(5), 50.

[ece35049-bib-0207] Terradas, J. , & Penuelas, J. (2011). Misleading ideas about top‐down and bottom‐up control in communities and the role of omnivores. Polish Journal of Ecology, 59(4), 849–850.

[ece35049-bib-0208] Thompson, R. M. , Hemberg, M. , Starzomski, B. M. , & Shurin, J. B. (2007). Trophic levels and trophic tangles: The prevalence of omnivory in real food webs. Ecology, 88(3), 612–617. 10.1890/05-1454 17503589

[ece35049-bib-0209] Tidemann S., & Gosler A. G. (Eds.) (2012). Ethno‐ornithology: Birds, indigenous peoples, culture and society. London, UK: Earthscan.

[ece35049-bib-0210] Tilman, D. , May, R. M. , Lehman, C. L. , & Nowak, M. A. (1994). Habitat destruction and the extinction debt. Nature, 371(6492), 65–66. 10.1038/371065a0

[ece35049-bib-0211] Tinbergen, N. (1989). The study of instinct. Oxford, UK: Oxford University Press.

[ece35049-bib-0212] Tori, G. M. , McLeod, S. , McKnight, K. , Moorman, T. , & Reid, F. A. (2002). Wetland conservation and ducks unlimited: Real world approaches to multispecies management. International Journal of Waterbird Biology, 25(2), 115–121.

[ece35049-bib-0213] Tylianakis, J. M. , Laliberté, E. , Nielsen, A. , & Bascompte, J. (2010). Conservation of species interaction networks. Biological Conservation, 143(10), 2270–2279. 10.1016/j.biocon.2009.12.004

[ece35049-bib-0214] UNESCO (2010). United Nations declaration on the rights of indigenous peoples. Paris, France: UNESCO.

[ece35049-bib-0215] UNESCO (2018). UNESCO policy on engaging with indigenous peoples. Paris, France: UNESCO Retrieved from http://unesdoc.unesco.org/images/0026/002627/262748e.pdf

[ece35049-bib-0216] Ungar, P. S. (1996). Relationship of incisor size to diet and anterior tooth use in sympatric Sumatran anthropoids. American Journal of Primatology, 38(2), 145–156. 10.1002/(SICI)1098-2345(1996)38:2<145:AID-AJP3>3.0.CO;2-Z 31918473

[ece35049-bib-0217] Ungar, P. S. , Grine, F. E. , & Teaford, M. F. (2006). Diet in early Homo: A review of the evidence and a new model of adaptive versatility. Annual Review of Anthropology, 35, 209–228.

[ece35049-bib-0218] Valiente‐Banuet, A. , Aizen, M. A. , Alcántara, J. M. , Arroyo, J. , Cocucci, A. , Galetti, M. , … Zamora, R. (2015). Beyond species loss: The extinction of ecological interactions in a changing world. Functional Ecology, 29(3), 299–307. 10.1111/1365-2435.12356

[ece35049-bib-0219] Vanni, M. J. (2002). Nutrient cycling by animals in freshwater ecosystems. Annual Review of Ecology and Systematics, 33, 341–370. 10.1146/annurev.ecolsys.33.010802.150519

[ece35049-bib-0220] Vermeij, G. J. (2012). The limits of adaptation: Humans and the predator‐prey arms race. Evolution, 66–7, 2007–2014. 10.1111/j.1558-5646.2012.01592.x 22759280

[ece35049-bib-0221] Visser, A. W. , Mariani, P. , & Pigolotti, S. (2012). Adaptive behaviour, tri‐trophic food‐web stability and damping of chaos. Journal of the Royal Society Interface, 9(71), 1373–1380. 10.1098/rsif.2011.0686 PMC3350732 22090284

[ece35049-bib-0222] Wall, D. H. , & Moore, J. C. (1999). Interactions underground: Soil biodiversity, mutualism, and ecosystem processes. BioScience, 49(2), 109–117.

[ece35049-bib-0223] West‐Eberhard, M. J. (1989). Phenotypic plasticity and the origins of diversity. Annual Review of Ecology and Systematics, 20(1), 249–278. 10.1146/annurev.es.20.110189.001341

[ece35049-bib-0224] Weyrich, L. S. , Duchene, S. , Soubrier, J. , Arriola, L. , Llamas, B. , Breen, J. , … Cooper, A. (2017). Neanderthal behaviour, diet, and disease inferred from ancient DNA in dental calculus. Nature, 544(7650), 357–361. 10.1038/nature21674 28273061

[ece35049-bib-0225] Whelan, C. J. , Wenny, D. G. , & Marquis, R. J. (2008). Ecosystem services provided by birds. Annals of the New York Academy of Sciences, 1134(1), 25–60. 18566089 10.1196/annals.1439.003

[ece35049-bib-0226] Wichmann, M. C. , Alexander, M. J. , Soons, M. B. , Galsworthy, S. , Dunne, L. , Gould, R. , Bullock, J. M. (2008). Human-mediated dispersal of seeds over long distances. Proceedings of the Royal Society B: Biological Sciences, 276(1656), 523–532. 10.1098/rspb.2008.1131 PMC2664342 18826932

[ece35049-bib-0227] Whelan, C. J. , Wenny, D. G. , & Marquis, R. J. (2008). Ecosystem services provided by birds. Annals of the New York Academy of Sciences, 1134(1), 25–60. 18566089 10.1196/annals.1439.003

[ece35049-bib-0228] Wilder, B. T. , Betancourt, J. L. , Epps, C. W. , Crowhurst, R. S. , Mead, J. I. , & Ezcurra, E. (2014). Local Extinction and Unintentional Rewilding of Bighorn Sheep (*Ovis canadensis*) on a Desert Island. PLoS ONE, 9(3), e91358 10.1371/journal.pone.0091358 24646515 PMC3960132

[ece35049-bib-0229] Williams, M. , Zalasiewicz, J. , Davies, N. , Mazzini, I. , Goiran, J. P. , & Kane, S. (2014). Humans as the third evolutionary stage of biosphere engineering of rivers. Anthropocene, 7, 57–63. 10.1016/j.ancene.2015.03.003

[ece35049-bib-0230] Wood, B. (2017). Evolution: Origin(s) of modern humans. Current Biology, 27(15), R767–R769. 28787610 10.1016/j.cub.2017.06.052

[ece35049-bib-0231] Wood, B. , & Lonergan, N. (2008). The hominin fossil record: Taxa, grades and clades. Journal of Anatomy, 212, 354–376. 10.1111/j.1469-7580.2008.00871.x 18380861 PMC2409102

[ece35049-bib-0232] Wood, B. , & Richmond, B. G. (2000). Human evolution: Taxonomy and paleobiology. Journal of Anatomy, 196, 19–60. 10.1046/j.1469-7580.2000.19710019.x PMC1468107 10999270

[ece35049-bib-0233] Worm, B. , & Paine, R. T. (2016). Humans as a hyperkeystone species. Trends in Ecology & Evolution, 31(8), 600–607. 10.1016/j.tree.2016.05.008 27312777

[ece35049-bib-0234] Wunder, S. , Angelsen, A. , & Belcher, B. (2014). Forests, livelihoods and conservation: Broadening the empirical basis. World Development, 64, S1–S11. 10.1016/j.worlddev.2014.03.006 PMC7220182 32405139

[ece35049-bib-0235] Zvelebil, M. , & Rowley-Conwy, P. (1984). Transition to farming in Northern Europe: A hunter-gatherer perspective. Norwegian Archaeological Review, 17(2), 104–128.

